# Pt(II) versus Pt(IV) in Carbene Glycoconjugate Antitumor Agents: Minimal Structural Variations and Great Performance Changes

**DOI:** 10.1021/acs.inorgchem.9b03683

**Published:** 2020-03-04

**Authors:** Alfonso Annunziata, Angela Amoresano, Maria Elena Cucciolito, Roberto Esposito, Giarita Ferraro, Ilaria Iacobucci, Paola Imbimbo, Rosanna Lucignano, Massimo Melchiorre, Maria Monti, Chiara Scognamiglio, Angela Tuzi, Daria Maria Monti, Antonello Merlino, Francesco Ruffo

**Affiliations:** † Dipartimento di Scienze Chimiche, Università di Napoli Federico II, Complesso Universitario di Monte S. Angelo, via Cintia 21, 80126 Napoli, Italy; ‡ CIRCC, via Celso Ulpiani 27, 70126 Bari, Italy; § Dipartimento di Chimica Ugo Schiff, Università di Firenze, Sesto Fiorentino, Florence 50019, Italy; ∥ ISUSCHEM, piazza Carità 32, 60134 Napoli, Italy

## Abstract

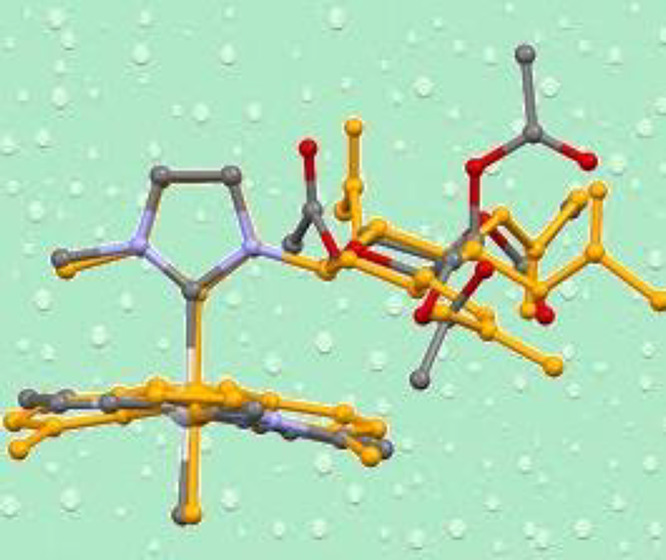

Octahedral Pt(IV) complexes (**2Pt–R**) containing a glycoconjugate carbene ligand were prepared and fully characterized. These complexes are structural analogues to the trigonal bipyramidal Pt(II) species (**1Pt–R**) recently described. Thus, an unprecedented direct comparison between the biological properties of Pt compounds with different oxidation states and almost indistinguishable structural features was performed. The stability profile of the novel Pt(IV) compounds in reference solvents was determined and compared to that of the analogous Pt(II) complexes. The uptake and antiproliferative activities of **2Pt–R** and **1Pt–R** were evaluated on the same panel of cell lines. DNA and protein binding properties were assessed using human serum albumin, the model protein hen egg white lysozyme, and double stranded DNA model systems by a variety of experimental techniques, including UV–vis absorption spectroscopy, fluorescence, circular dichroism, and electrospray ionization mass spectrometry. Although the compounds present similar structures, their in-solution stability, cellular uptake, and DNA binding properties are diverse. These differences may represent the basis of their different cytotoxicity and biological activity.

## Abstract 2

An unprecedented direct comparison between the biological properties of Pt compounds with different oxidation states and almost indistinguishable structural features was performed. The reasons of the diverse activity and selectivity have been investigated through several techniques by evaluating structural and stability properties, cellular uptake, and the interaction with macromolecules.

## Introduction

**Figure 1:**
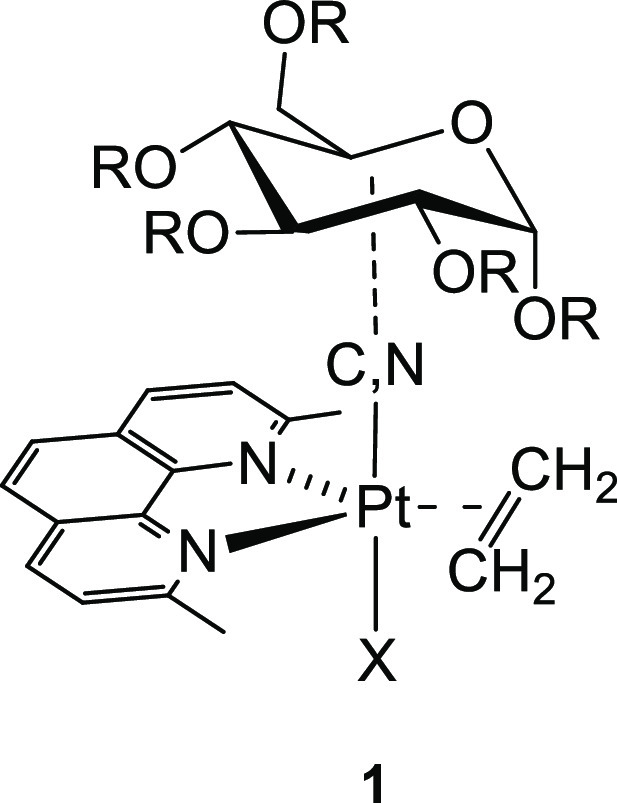
Sketch of the glycoconjugate *tbp* platinum(II) complex (**1**).

The improvement of the anticancer performance of metal-based agents is an important task of modern chemistry.^1^ Among the numerous strategies aimed at enhancing both activity and selectivity of these molecules, conjugation with biologically active molecular fragments targeting tumor cells is very promising.^2−8^ In this context, our group has recently investigated Pt(II) complexes in 18 e– trigonal bipyramidal geometry (*tbp*) containing a sugar-based axial ligand (**1** in Figure 1).^9−12^

**Figure 2:**
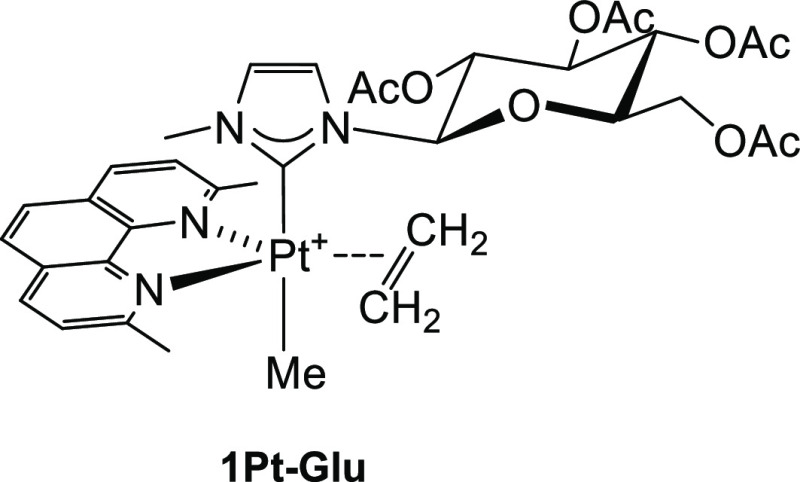
Formula of the glucoconjugate *tbp* platinum(II) complex **1Pt–Glu**.

This choice simultaneously exploited: (i) the importance of the oxidation state (II), (ii) the stability of the coordinative saturation, and (iii) the possible target recognition of the sugar fragment mediated by the Warburg effect.^13,14^ During these studies, interesting results have been obtained with a cationic complex containing a glucoconjugate carbene (**1Pt–Glu** in Figure 2) that showed a cytotoxic effect on cancer cells 2 orders of magnitude higher than cisplatin. Moreover, a significant selectivity was found for cancer cells (SVT2 and A431) with respect to immortalized cells (BALB/c-3T3 and HaCaT).^11^

Other evidence that disclosed straight consequences upon small variations of the coordination environment accompanied this gratifying result: the simple substitution glucose → galactose (epimers at C4) or the removal of the protecting groups rendered the complex significantly less effective. The results were framed in the light of the key-role played by the sugar portion, whose nature and polarity can affect internalization, the metallodrug cellular pathway, and target recognition.^15−17^

**Figure 3:**
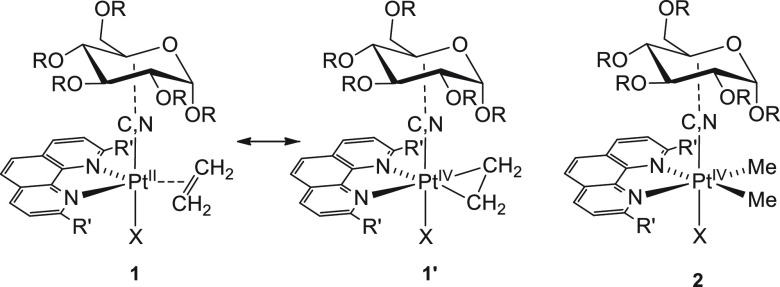
Structural analogy between trigonal bipyramidal (**1**) and octahedral (**2**) complexes This assumption suggested the design of new cationic complexes of Pt(IV) containing two methyl ligands in equatorial positions and the glycoconjugate carbene in one axial site (**2Pt–R** in Figure 4).

Since fine modulations in the chemical properties of the investigated glucoconjugated Pt(II)-based carbene compounds are associated with significant differences in their biological properties, we have planned to verify the consequences of the change in the oxidation state of Pt on the biological activity of these compounds, preparing Pt(IV) analogues. This study allows a rather rare “true” comparison within the Pt(II)/Pt(IV) analogues.^18−21^ In fact, while literature data widely demonstrated that Pt(IV) pro-drugs are competitive with Pt(II) agents,^22−37^ it should be underlined that the transition to the higher oxidation state involves large structural variations (e.g., from square-planar to octahedral geometry), which makes a direct comparison between analogous Pt(IV) and Pt(II) compounds less homogeneous and more difficult to interpret. Therefore, the availability of species in the two different states of oxidation, but with an overlapping coordination environment, would provide an unprecedented opportunity to fill this gap. Actually, the platinum(II) complexes **1** in *tbp* geometry are expected to share considerable structural aspects with an octahedral platinum(IV) species of type **2** (Figure 3), where two methyl groups substitute ethylene: the structural analogy becomes even more evident considering that the strong Pt-to-ethylene π-backdonation in **1**, typical of trigonal bipyramidal complexes,^38^ results in a partial sp^2^ → sp^3^ rehybridization of the alkene carbons, and the whole structure approximates to a Pt(IV)-cyclometallate (**1′**).

In line with the premises, NMR spectroscopy and X-ray diffraction disclosed the stringent structural analogy with the corresponding trigonal bipyramidal Pt(II) species (**1Pt–R**) complexes.^11^ Instead, the biological studies revealed deep changes in the cytotoxic properties: **2Pt–Glu** as well as the other Pt(IV) complexes did not show satisfactory activity and selectivity toward cancer cells, confirming that minimal structural variations heavily affect the performance. These conflicting results were a stimulus for a comparative study between **1Pt–Glu** and **2Pt–Glu** to gain insights about the effects of the formal difference in the oxidation state and to access more information about the mechanisms of action that strongly enhance the biological performance of the former one.

**Figure 4:**
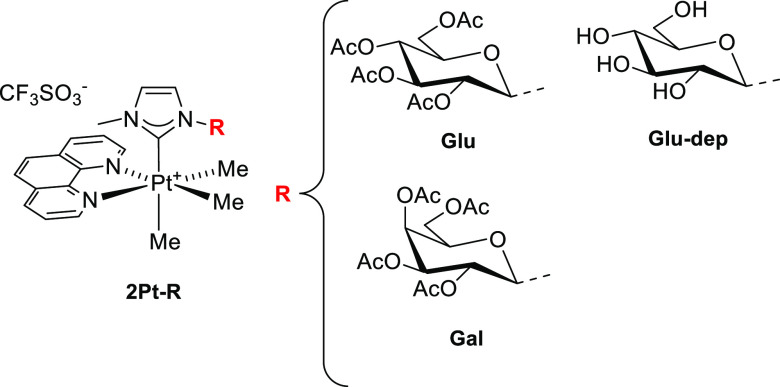
Formula of the glicoconjugate octahedral platinum(IV) complexes **2Pt–R**. Labeling of the complexes: Glu = glucose, Gal = galactose, and dep = deprotected.

Hence, this work reports synthesis, spectroscopic, and structural characterization of octahedral Pt(IV) complexes (**2Pt–R**; Figure 4), along with a thorough comparative study of their chemical stability in different experimental conditions and of biological properties, comprising cytotoxic activity, cellular uptake, interaction with DNA and proteins.

## Results

### Synthesis and Structural Characterization of Complexes **2Pt–R**

**Scheme 1:**
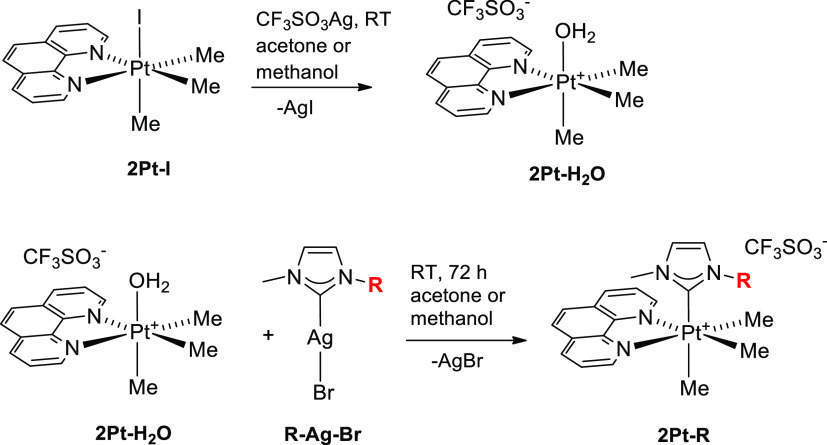
Synthesis of Complexes of Type **2Pt–R**

Scheme 1 displays the synthesis of complexes **2Pt–R**. The aquo-precursor **2Pt–H**_**2**_**O** was obtained by suspending **2Pt–I** in a solution containing silver triflate in acetone or methanol. The precipitated AgI was filtered off, and an equivalent of the appropriate silver carbene **R–Ag–Br** was added to the solution. The mixture was stirred for 3 days in acetone or in methanol. The precipitated AgBr was removed by filtration, and the **2Pt–R** complexes were crystallized, either with the hydroxyls in acetylated form, when the reaction was carried out in acetone, or deprotected, when the solvent was methanol. In this second case, the acetyl groups undergo transesterification catalyzed by the Lewis acidity of the Ag(I) ion present in the reaction system.

The complexes display octahedral geometry with the equatorial plane defined by the bidentate ligand 1,10-phenanthroline (phen) and two methyls, while the axial positions are occupied by the carbene and a third methyl. The presence of phen represents the second minimal difference with respect to **1Pt–R** complexes, which contain 2,9-dimethyl-1,10-phenanthroline (dmphen), which is necessary to ensure the stability of the *tbp* geometry. Attempts to introduce dmphen in type **2** complexes did not produce satisfactory results.

**Figure 5:**
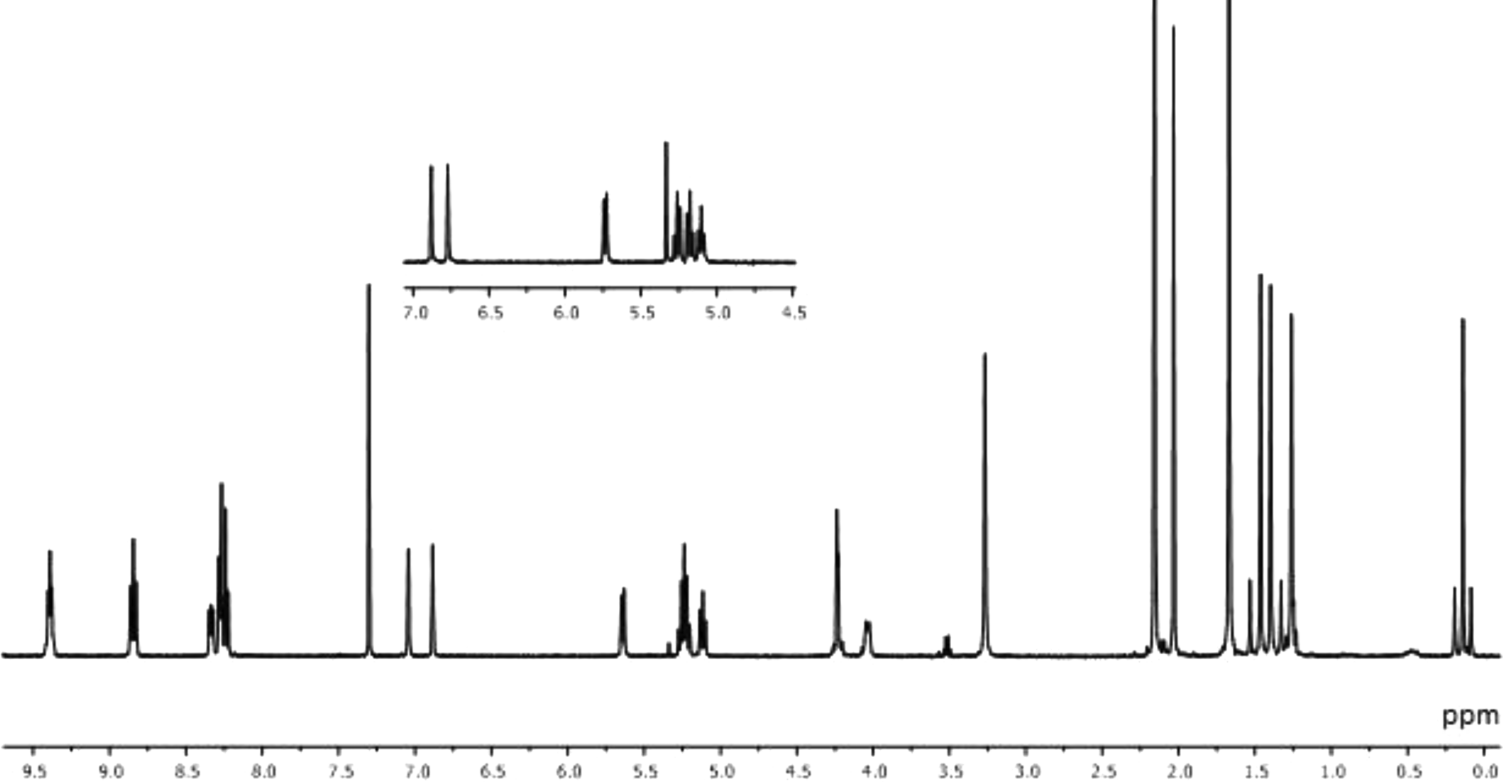
^1^H NMR spectrum of **2Pt–Glu** in CDCl_3_ at 298 K. The inset shows the corresponding portion of **1Pt–Glu**.

The complexes were characterized by NMR spectroscopy (Figures S1–S4) and X-ray diffraction (Table S1 and Figures S5–S9). The following observations contributed to the characterization of the complexes: a singlet close to δ 0 and two singlets around δ 1.5, attributable, respectively, to the axial methyl and to the two nonequivalent equatorial methyls, with the satellites due to coupling to ^195^Pt; the multiplets of the sugar protons with the expected coupling constants; two singlets at ca. δ 7 for the protons riding on the carbene ligand. The signals were practically coincident with the corresponding ones of **1Pt–R** complexes (Figure 5). The Pt–C (carbene) resonances were found at the expected frequencies in the carbon spectra (^1^*J*_Pt–C_ ca. 600 Hz).

**Figure 6:**
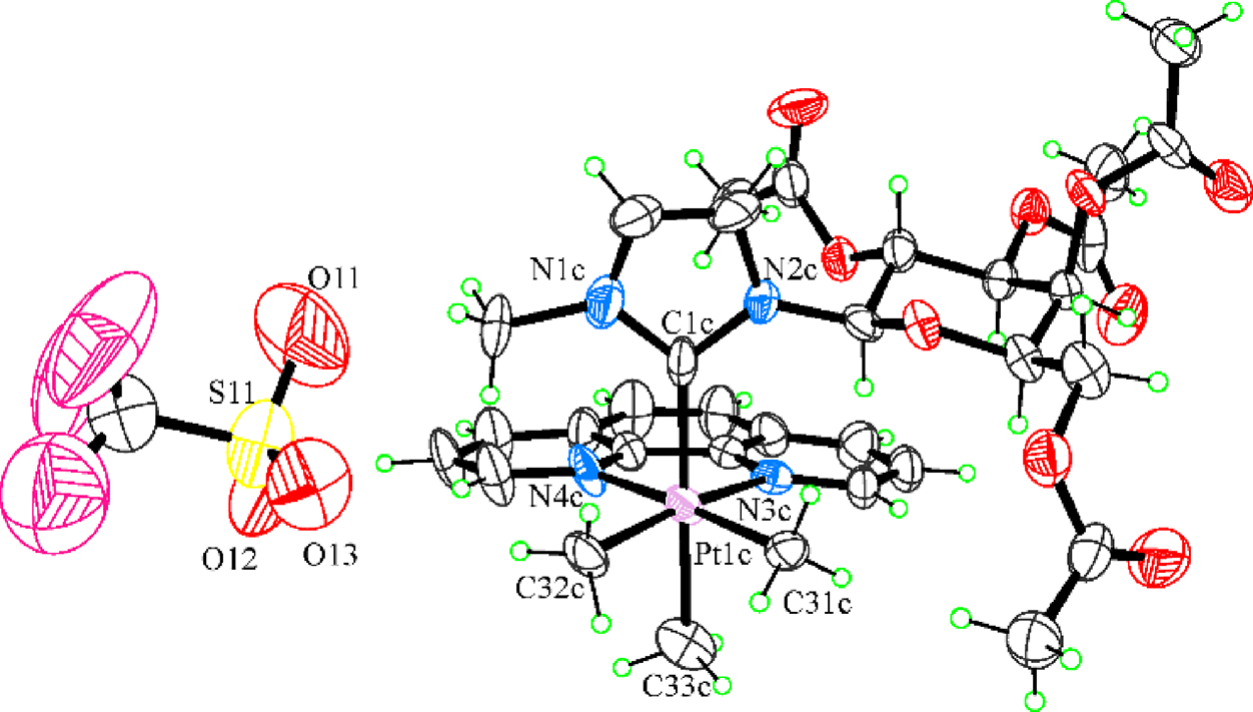
ORTEP view of one of the four independent molecules of **2Pt–Gal**. Thermal ellipsoids are drawn at a 30% probability level.

The single crystal X-ray analysis confirmed that compound **2Pt–Gal** contains a cationic carbene complex of Pt(IV) with CF_3_SO_3_^–^ (triflate) as counterion. The structure of **2Pt–Gal** is reported in Figure 6. Details of the structural analysis are reported in the Supporting Information.

The compound crystallizes in the triclinic *P1* space group with four independent pairs of cations and anions in the unitary cell. All bond lengths and angles are in the expected range. No significative differences in the geometric parameters were found between the four independent pairs of molecular ions; only one pair is reported in Figure 6. The Pt atom adopts a fairly regular octahedral geometry with the bidentate phen ligand and two methyl groups in the equatorial plane. The two Pt–Me distances (2.05(2) and 2.07(2) Å) are in line with previous literature data.^39^ The axial positions are occupied by the central carbon atom of the carbene ligand and by a third methyl group. The crystal structure of **2Pt–Gal** revealed a close analogy with **1Pt–Glu** reported previously.^11^ Despite the different oxidation state of platinum, the coordination environment in **2Pt–Gal** overlaps well with that observed in the structure of **1Pt–Glu**. The glycoconjugate carbene groups also overlap well, apart from the changed axial or equatorial position in the ring. The galactosyl group at the N2 atom is in the usual chair conformation with three equatorial and one axial substituents. At variance with **1Pt–Glu**, a not flat shape is adopted by the galactosyl group due to the axial substituent that is placed far away from the phen ligand plane to avoid steric effects. A less evident bowlike distortion of the phen bidentate ligand is observed with respect to **1Pt–Glu**, with the dihedral angle between the mean planes of the outer rings ranging from 8(2)° to 11(2)° in the four independent cations. In the crystal, the triflate anions are placed in the neighborhood of Pt(IV), with a mean Pt···O (triflate) distance of 5.91(3) Å. The crystal packing is dominated by electrostatic interactions and is also stabilized by weak C–H···O interactions.

### In-Solution Studies

**Figure 7:**
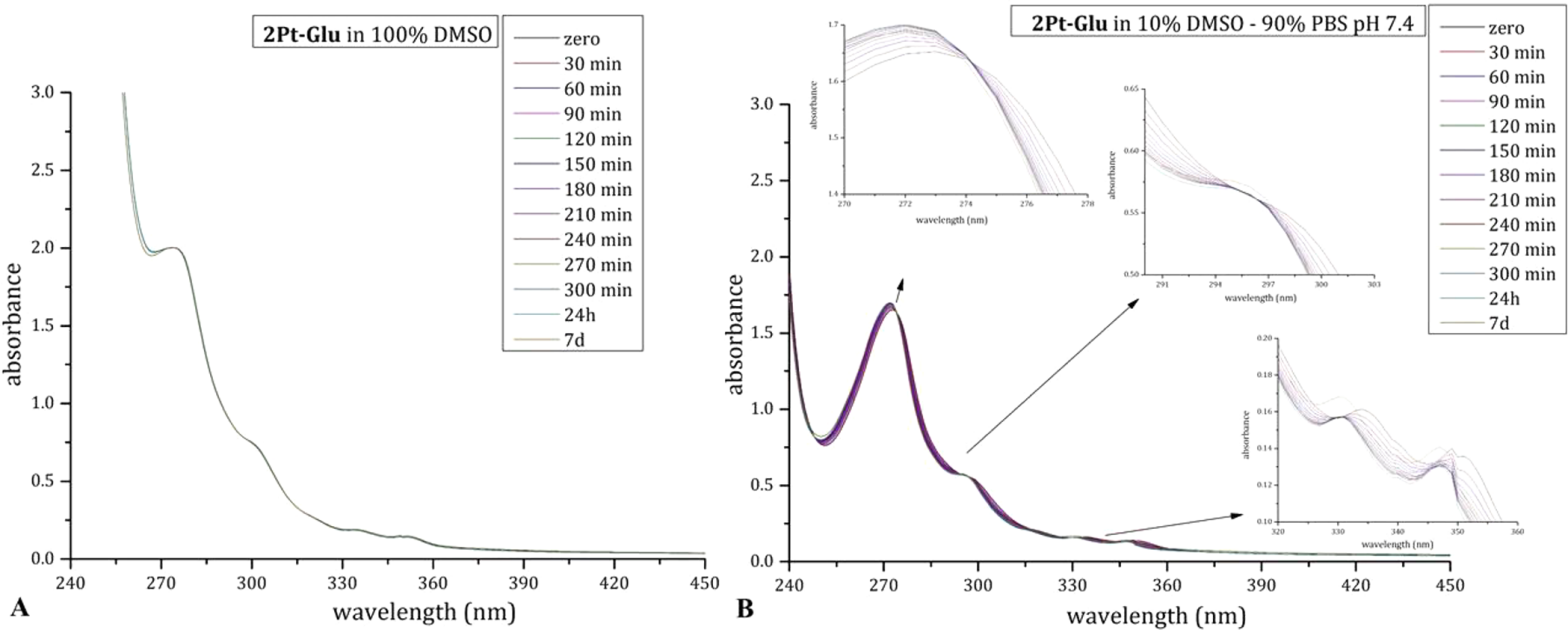
Time course UV–vis spectra of 50 μM **2Pt–Glu** in 100% DMSO (A) and 10% DMSO/90% PBS (pH 7.4) (B). In (B), details of the isosbestic points are also shown.

Relevant aspects of the in-solution behavior of **2Pt–Glu** were studied by ^1^H NMR and UV–vis absorption spectroscopy in comparison to **1Pt–Glu**.^11^ UV–vis spectra of **2Pt–Glu** were recorded in aqueous media (10% DMSO/90% PBS (pH 7.4) and 50% DMSO/50% PBS (pH 7.4)) and pure DMSO and reported in Figures 7A,B and S10. Interestingly, **1Pt–Glu** and **2Pt–Glu** showed very different stabilities. **1Pt–Glu** is stable in aqueous media, while it exchanges ethylene and dmphen ligands for a solvent molecule in pure DMSO.^11^ On the contrary, **2Pt–Glu** does not show appreciable changes in pure DMSO, while it undergoes spectral changes with time in aqueous media. The presence of isosbestic points in the spectral profiles of **2Pt–Glu** in mixed DMSO/PBS solutions (Figures 7B and S10) confirmed the occurrence of a ligand exchange process.

**Scheme 2:**
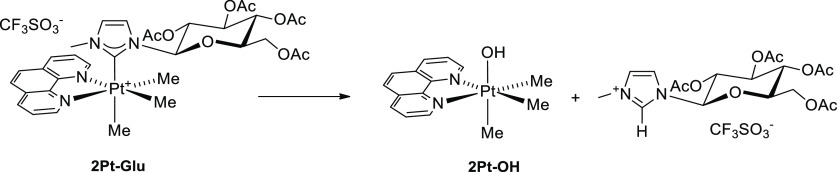
Hydrolysis of **2Pt–Glu**

Similar results were observed by ^1^H NMR. The analysis of the NMR spectra in 10% DMSO/90% PBS (pH 7.4) indicates that **2Pt–Glu** undergoes hydrolysis of the carbene moiety, yielding a hydroxyl Pt species (**2Pt–OH** in Scheme 2) and the imidazolinium salt.^40−42^ The process is complete within 24 h (Figure S11). The two products were identified by comparing their NMR spectra with those of authentic samples. An influence of pH was observed; as in D_2_O, the same process occurred slowly. On the other hand, **1Pt–Glu** is stable over days under the same experimental conditions.^11^

### Interaction with DNA

The interaction of **2Pt–Glu** with DNA was studied by fluorescence, circular dichroism, electrospray ionization mass spectrometry, and ^1^H NMR, in comparison to that of **1Pt–Glu**.^11^

The binding of **2Pt–Glu** to calf-thymus DNA (ctDNA) was first evaluated by using the ethidium bromide (EtBr) displacement fluorescence assay (Figure S12). Results of the fluorescence assay indicate that **2Pt–Glu** does not displace EtBr from the ctDNA major groove, as is observed also in the case of **1Pt–Glu** and differently from cisplatin and the dmphen ligand.^11^

**Figure 8:**
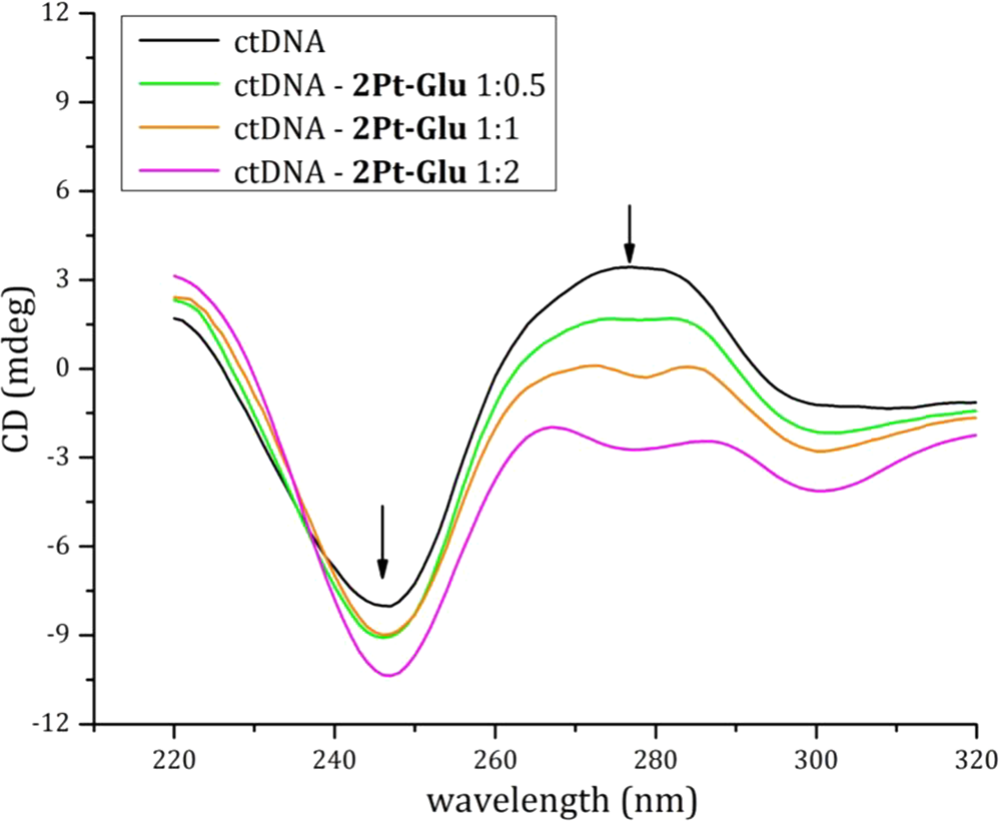
CD spectra of ctDNA (200 μM in 0.01 M ammonium acetate buffer, pH 7.5) in the absence (black line) and in the presence of **2Pt–Glu** with different DNA to metal molar ratios (1:0.5 green line, 1:1 orange line, and 1:2 purple line).

Then, CD spectra of ctDNA in the presence of **2Pt–Glu** in different molar ratios were registered and compared to the spectrum of drug-free DNA (Figure 8).

The CD spectra of ctDNA present the typical features of the right-handed B form of DNA, in agreement with literature data.^11^ In the presence of **2Pt–Glu**, the intensities of both positive and negative bands shift to lower ellipticity values, as is observed for **1Pt–Glu**.^11^

**Figure 9:**
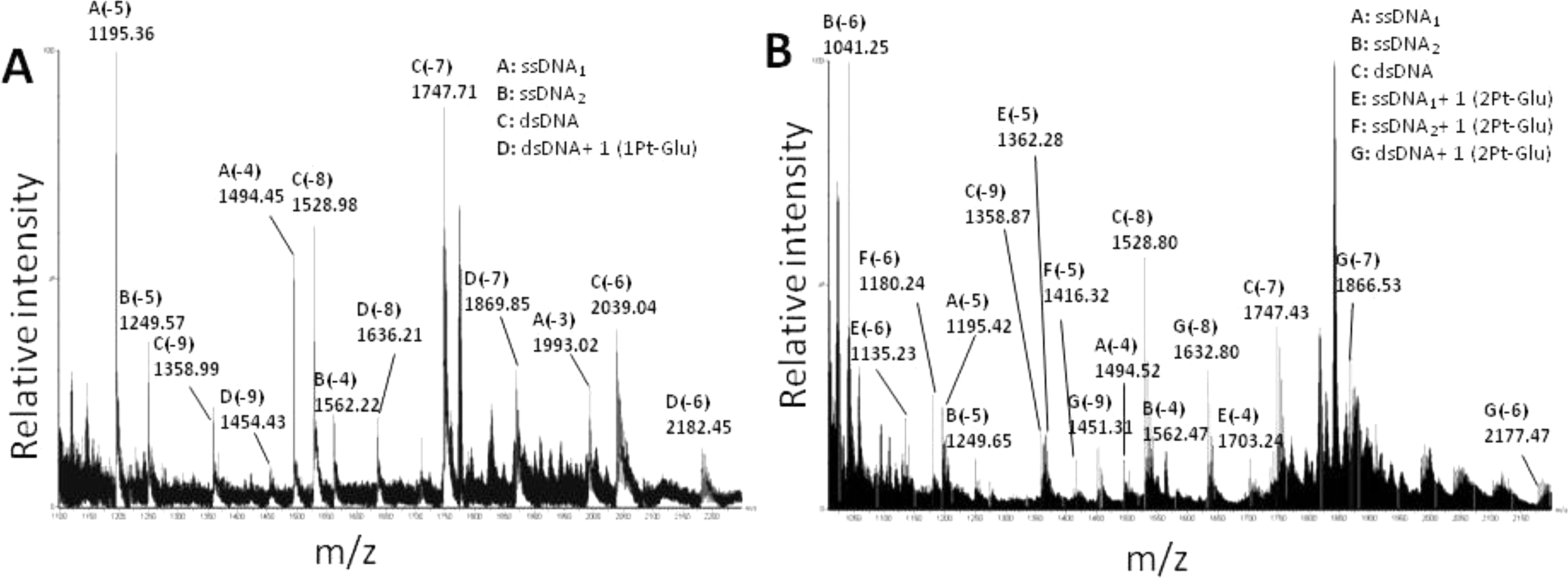
ESI-MS spectra of dsDNA incubated with (A) **1Pt–Glu** and (B) **2Pt–Glu**.

Successively, to shed light on the binding of the two compounds to a DNA model system at molecular level, the binding of **1Pt–Glu** and **2Pt–Glu** to a 20 mer double stranded oligonucleotide (dsDNA) was investigated by electrospray mass spectrometry. ESI-MS spectra of **1Pt–Glu** and **2Pt–Glu** are reported in Figure 9A,B, respectively, and all signals detected are summarized in Table 1. In both cases, one molecule of each Pt compound binds the dsDNA, as demonstrated by the presence of the species at molecular weights of 13 096.53 ± 2.20 Da and 13 071.34 ± 0.87 Da (Table 1), ascribable to dsDNA bound to one molecule of **1Pt–Glu** and one of **2Pt–Glu**, respectively (Figure 9).

**Table 1:** Results of ESI-MS Analysis of Species Formed upon Reaction of DNA with **1Pt–Glu** and **2Pt–Glu**^a^

metal complex	signal (*m*/*z*)	signal charge	exp MW (Da)	theoretical MW (Da)	species
**1Pt–Glu**	1195.36	A (−5)	5982.92 ± 0.80	5983.9	ssDNA_1_
	1494.45	A (−4)			
	1993.02	A (−3)			
	1249.57	B (−5)	6254.45 ± 0.50	6255.1	ssDNA_2_
	1562.22	B (−4)			
	1358.99	C (−9)	12240.33 ± 0.30	12239	dsDNA
	1528.98	C (−8)			
	1747.71	C (−7)			
	2039.04	C (−6)			
	1454.43	D (−9)	13096.53 ± 2.20	13097.8	dsDNA + **1** (**1Pt–Glu**)
	1636.21	D (−8)			
	1869.85	D (−7)			
	2182.45	D (−6)			
**2Pt-Glu**	1195.42	A (−5)	5982.15 ± 0.03	5983.9	ssDNA_1_
	1494.52	A (−4)			
	1041.25	B (−6)	6253.58 ± 0.27	6255.1	ssDNA_2_
	1249.65	B (−5)			
	1562.47	B (−4)			
	1358.87	C (−9)	12238.82 ± 0.27	12239	dsDNA
	1528.80	C (−8)			
	1747.43	C (−7)			
	1135.23	E (−6)	6817.08 ± 0.46	6816.15	ssDNA_1_ + **1** (**2Pt–Glu**)
	1362.28	E (−5)			
	1703.24	E (−4)			
	1180.24	F (−6)	7087.04 ± 0.42	7087.35	ssDNA_2_ + **1** (**2Pt–Glu**)
	1416.32	F (−5)			
	1451.31	G (−9)	13071.34 ± 0.87	13071.3	dsDNA + **1** (**2Pt–Glu**)
	1632.80	G (−8)			
	1866.53	G (−7)			

a The *m*/*z* values detected in MS spectra and their relative charges as well as experimental (exp) and theoretical (theor) monoisotopic mass values and the corresponding ion species are reported. dsDNA = double stranded DNA; ssDNA = single stranded DNA.

These data suggest that the Pt compounds share common features in binding the dsDNA. Moreover, the behavior of the two molecules is different from that observed in the reaction of the same DNA model system with cisplatin. Indeed, when the dsDNA was incubated with cisplatin, under the same experimental conditions, up to three cisplatin molecules bound the dsDNA. However, upon binding, each cisplatin molecule lost both Cl^–^ ligands, and some of them were replaced by other fragments (i.e., acetate ions, Figure S13 and Table S1).

From these data, it appears that **2Pt–Glu** is more stable in aqueous media in the presence of DNA. ESI spectra also revealed a difference in the binding affinity of **1Pt–Glu** and **2Pt–Glu** toward ssDNA. In the case of **2Pt–Glu**, two additional peaks were detected at 6817.08 ± 0.46 and 7087.04 ± 0.42 Da (Figure 9B). These peaks are indicative of a partial binding of **2Pt–Glu** to ssDNA. This ssDNA is in equilibrium with dsDNA and is already detectable in the ESI spectrum in the absence of **2Pt–Glu** (data not shown). No additional peaks were observed in the ESI spectra collected upon incubation of **1Pt–Glu** with dsDNA under the same experimental conditions.

**Figure 10:**
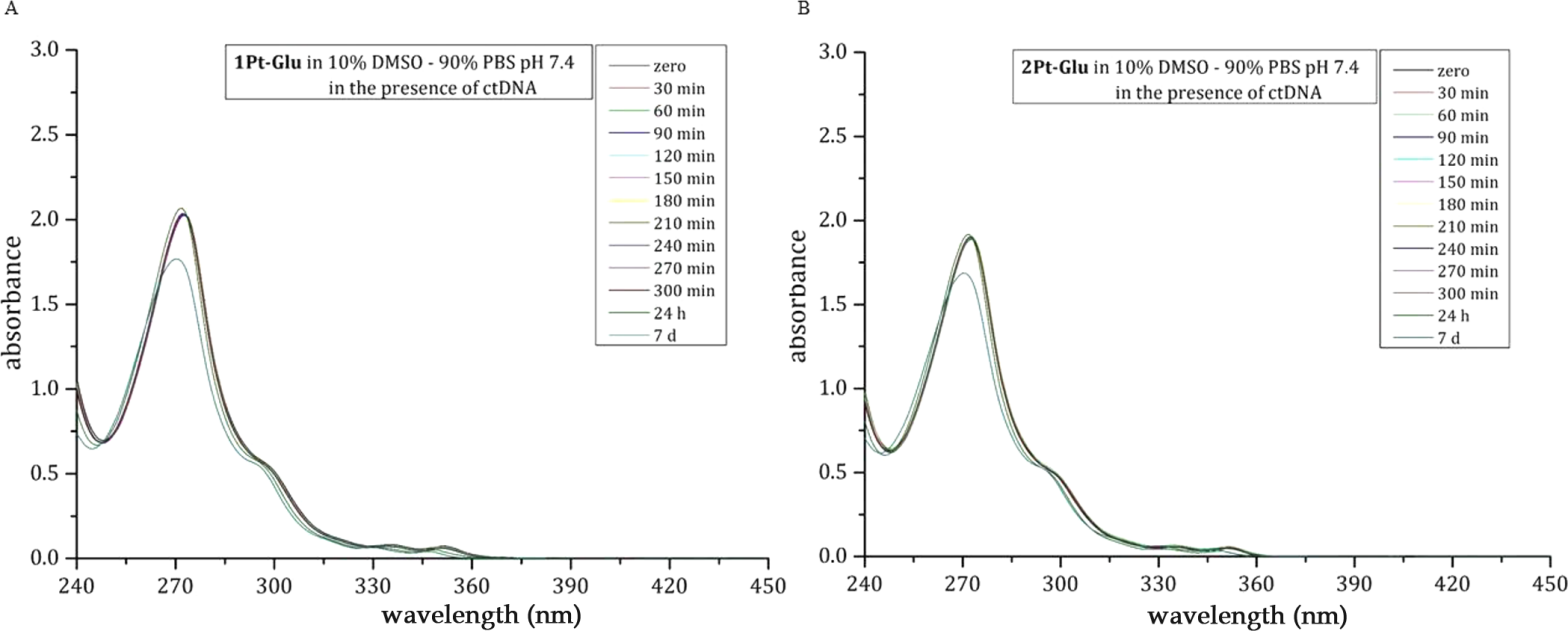
Time course UV–vis spectra of 50 μM **1Pt–Glu** (A) and 50 μM **2Pt–Glu** (B) in 10% DMSO/90% PBS (pH 7.4) in the presence of ctDNA.

To further confirm that DNA was able to increase the stability of the Pt compounds, a time course in the UV–vis spectra of **2Pt–Glu** and **1Pt–Glu** in the presence of DNA was performed (Figure 10). The UV–vis spectra remain unchanged during the time course, with a minimal shift of the maximum absorption peak from 273 to 270 nm observed only after 7 days. This experiment confirmed that **2Pt–Glu** is more stable in aqueous media in the presence of DNA.

Finally, to obtain further insights into the reactivity of the complexes with DNA, the interaction of **1Pt–Glu** and **2Pt–Glu** with 2-deoxyguanosine monophosphate (dGMP) was investigated by ^1^H NMR. **1Pt–Glu** and **2Pt–Glu** were incubated at 37 °C for up to 2 weeks in PBS 90% (pH 7.4)/DMSO 10%, and spectra were recorded at different times of incubation. Under these conditions, the coordination of guanosine was not observed.

### Interactions with Proteins

**Figure 11:**
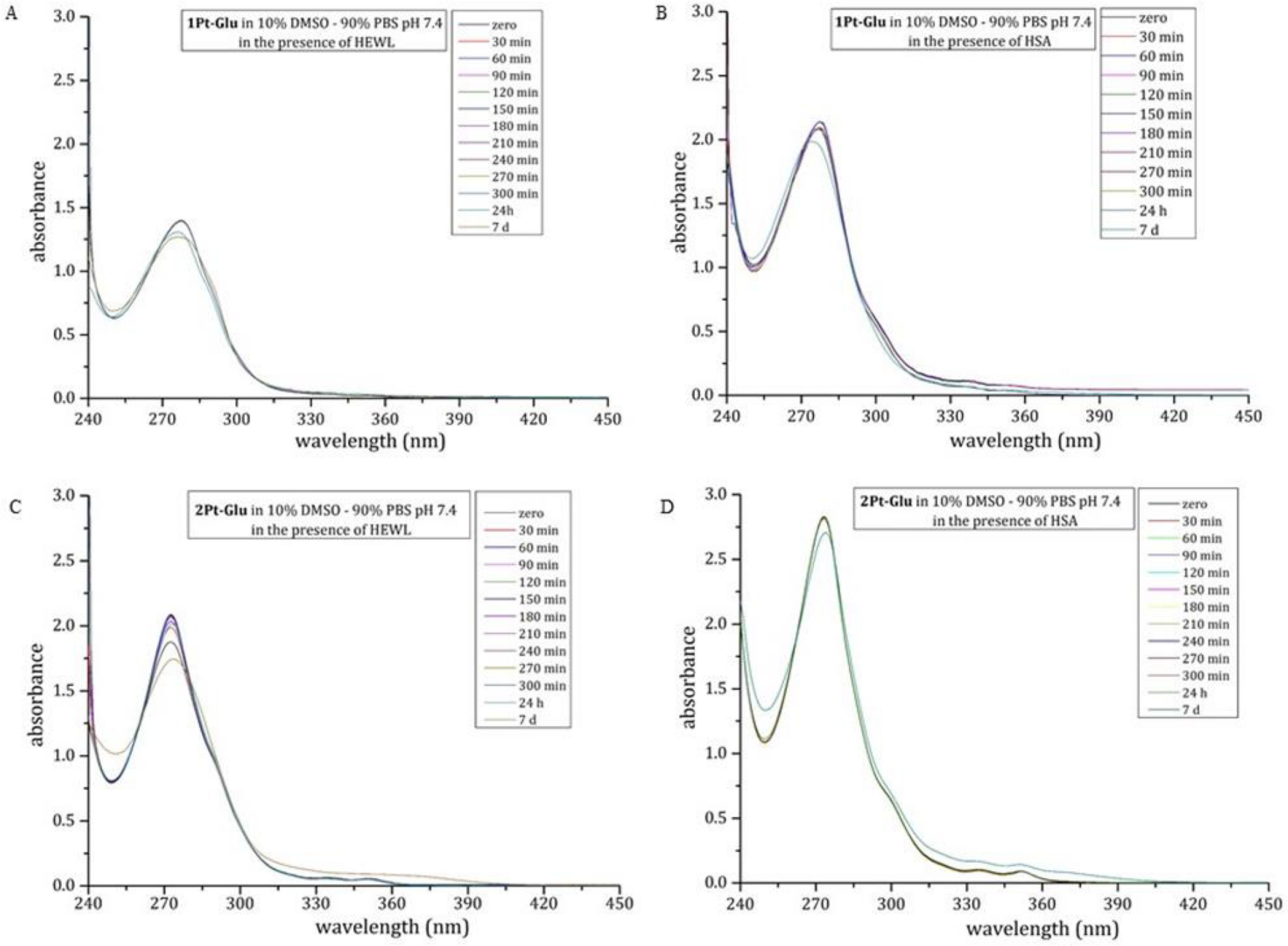
Time course UV–vis spectra of 50 μM **1Pt–Glu** (A, B) and 50 μM **2Pt–Glu** (C, D) in 10% DMSO/90% PBS (pH 7.4) in the presence of HEWL (A, C) and HSA (B, D) at a 1:3 protein to metal molar ratio.

The possible interaction of **1Pt–Glu** and **2Pt–Glu** with the model protein hen egg white lysozyme (HEWL) and with human serum albumin (HSA) was then studied by UV–vis absorption spectroscopy and circular dichroism. UV–vis spectra of the two compounds in the absence and in the presence of HEWL and HSA were collected over 7 days under different experimental conditions (Figures 11 and S14).

The analysis of the spectral profiles shows that both **1Pt–Glu** and **2Pt–Glu** are rather stable in the presence of the two proteins. The comparison between these spectra and those collected for the compounds in the absence of the proteins (Figure 7) indicates that the incubation of **2Pt–Glu** with HEWL or with HSA increases the in-solution stability of this metallodrug in the aqueous media, in agreement with what is observed in the presence of DNA.

**Figure 12:**
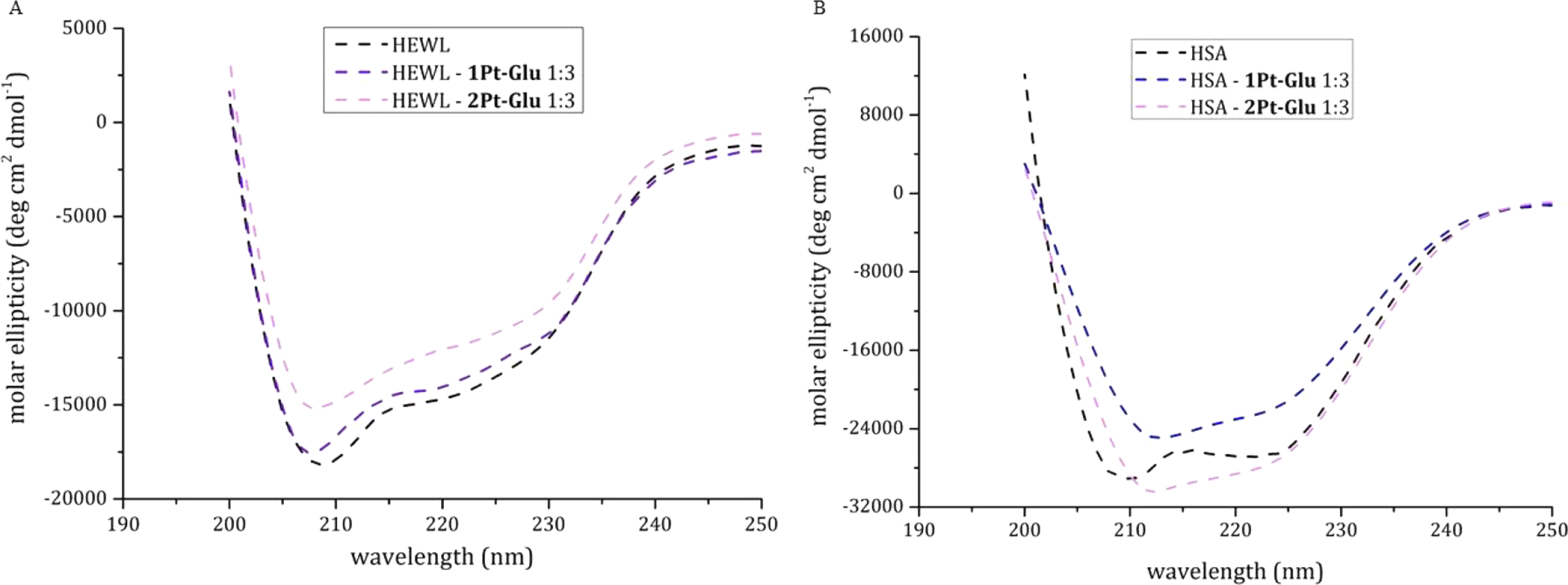
Far UV-CD spectra of HEWL (A) and HSA (B) incubated for 24 h in the presence of **1Pt–Glu** (dark purple/blue dashed curve/spectrum) and **2Pt–Glu** (light purple dashed curve/spectrum) in 10% PBS (pH 7.4) at a 1:3 protein to metal molar ratio. Free protein is represented by black dashed curves/spectra. (Protein concentration 0.10 mg/mL).

To evaluate in detail the potential interaction of **1Pt–Glu** and **2Pt–Glu** with HEWL and HSA, the secondary structure content of the two proteins was evaluated by CD spectroscopy at increasing concentrations of the metal compounds. Far UV-CD spectra were collected upon 24 h of incubation at room temperature. CD spectra reported in Figure 12 show a decrease of the molar ellipticity at increasing concentrations of **1Pt–Glu** and **2Pt–Glu** for both HEWL and HSA. This is indicative of a potential binding of the Pt compounds to the proteins.

### Cytotoxicity and Cellular Uptake Experiments

Finally, in order to inspect any biological difference between **1Pt–Glu** and **2Pt–Glu**, the cytotoxicity of **2Pt–Glu** and its derivatives was assessed by the MTT assay. The same panel of cells used to study the biological activity of **1Pt–Glu** were used. Cells were incubated with increasing concentrations of the Pt compounds, and then, cell survival was evaluated after 48 h of incubation. The IC_50_ values are reported in Table 2, and the results previously obtained with **1Pt–Glu** and cisplatin are reported for comparison.^11^ No cytotoxic activity was reported for **2Pt–Gal**, up to 125 μM, evidencing a close analogy with its analogue **1Pt–Gal**, which was considerably less active and selective than **1Pt–Glu**.^11^ These data suggest that the cytotoxic activity of these classes of compounds (**1Pt–R** and **2Pt–R**) is strictly dependent on the sugar portion and very sensible upon its small variations. Surprisingly, **2Pt–Glu** showed a completely different behavior with respect to **1Pt–Glu**, as it was about 100 times less toxic than **1Pt–Glu** and it completely lost its selectivity for the cancer cells analyzed. As **2Pt–Glu** can be hydrolyzed in aqueous buffer to form **2Pt–OH**, the latter compound was tested for its cytotoxicity. **2Pt–OH** was found to be more toxic than **2Pt–Glu** on immortalized cells, but still no selectivity was observed.

**Table 2:** IC_50_ Values (μM) Obtained for Pt Compounds on A431, SVT2, BALB/c-3T3, and HaCaT Cells after 48 h of Incubation^a^

cell line	**2Pt–Glu**	**2Pt–Glu–dep**	**2Pt–Gal**	**2Pt–OH**	**1Pt–Glu**(11)	cisplatin^11^
HaCaT	43.8 ± 4.3	55 ± 9	>125	13.6 ± 1.6	13 ± 1.7	6.6 ± 0.3
A431	43.8 ± 2.8	41.8 ± 1.7	>125	14.9 ± 0.6	0.40 ± 0.01	39 ± 12
BALB/c-3T3	196 ± 14	59.6 ± 2.6	>125	7.2 ± 0.7	6.3 ± 0.4	240 ± 47
SVT2	176 ± 13	122 ± 5	>125	6.4 ± 0.6	0.65 ± 0.07	195 ± 7

a The IC_50_ values for **1Pt–Glu** and cisplatin are from ref (11).

Thus, a different mechanism of action occurring between the two drugs was hypothesized, and their uptake in A431 cells was analyzed. Cancer cells were incubated with either **1Pt–Glu** or **2Pt–Glu** at the concentrations needed to reach the IC_50_ values. Cisplatin was used as a reference. After a 48 h incubation, the Pt content was measured by ICP-MS. The amount of Pt uptake in A431 cells was 0.65 ± 0.15% for **1Pt–Glu** with respect to 0.39 ± 0.09% for **2Pt–Glu** and 0.79 ± 0.25% for cisplatin. These data indicate that in the case of **1Pt–Glu** the percentage of Pt internalized by the cells is about 2 times higher than that found in the case of the cells treated with **2Pt–Glu**. Noteworthy, in the case of **1Pt–Glu**, the amount of Pt needed to reach the IC_50_ is about 100 times lower than that needed with **2Pt–Glu**.

### Attempts to Reduce **2Pt–Glu** In Vitro

To explain the decrease in the cytotoxic activity observed for **2Pt–R** compared to **1Pt–R**, we focused on the activation of Pt(IV) prodrugs, which are known to undergo a reduction in the biological environment, releasing the Pt(II) active species.^30,43^ In vitro reduction was attempted using ascorbic acid and glutathione, which are the most abundant reducing agent in intracellular media. **2Pt–Glu** (1 mM) was incubated at 37 °C in 90% PBS (pD 7.4)/10% DMSO with different excesses (2–25 mM) of the reducing agent, and ^1^H NMR spectra were recorded at different times of incubation. In these conditions, no sign of reduction was observed for **2Pt–Glu** or for its hydrolysate form **2Pt–OH** (Scheme 2). Furthermore, no coordination of glutathione ligands was observed, despite the known ability of sulfur ligands to coordinate in the axial positions of coordinatively saturated complexes.^44^

The lack of chemical reduction of **2Pt–Glu** and **2Pt–OH** is in agreement with the positions of their reduction peaks in the cyclic voltammograms, which are significantly more negative than those of Pt(IV) complexes containing chloride, acetate, or hydroxide ligands.^45^ In DMSO, peaks of reduction were observed at −1.54 and −1.89 V for **2Pt–Glu** and −1.40, −1.59, and −1.89 V for **2Pt–OH** (Figures S15 and S16 and Table S3). This trend is in accord with those observed for organometallic Pt(IV) compounds containing aryl substituents, which do not undergo chemical reduction as well.^46^

## Discussion

Recently, we described novel five-coordinate Pt(II) compounds bearing glycoconjugate carbene ligands, which were characterized and evaluated as potential anticancer compounds in vitro.^11^ One of these agents (**1Pt–Glu** in Figure 2) showed promising in vitro cytotoxic activity and high selectivity toward malignant cells. Since fine variations in its structure were associated with significant differences in the biological properties, we were also intrigued to assess the influence of the oxidation state of the metal, another factor of great impact on the biological properties of the complexes. The starting point of the present work was that the effect of the metal oxidation state could be assessed only with other structural aspects being equal. This perspective cannot be easily realized if the comparison regards the Pt(II)/Pt(IV) couple, by virtue of the different coordination geometries that these ions typically assume in their complexes. In this work, the target was achieved by exploiting the analogy between the cyclopropametallate fragment, typical of the Pt(II)–ethene bond, and the (bis-methyl)Pt(IV) moiety. The introduction of these motifs in the equatorial plane of trigonal bipyramidal and octahedral species (Figure 3) offers the unprecedented possibility of making a homogeneous comparison between Pt(II) and Pt(IV) compounds (**1Pt–R** and **2Pt–R**, respectively) regarding spectroscopic and structural features, solution stability, biological properties, and the ability to interact with macromolecules.

The experimental data illustrated in this work disclose relevant analogies and differences between the two classes. In full agreement with the initial expectations, their structures are nearly superimposable, and the close similarity of the NMR signals (Figure 5) confirms the similitude between the coordination environments. This returns a practically coincident molecular volume, despite the different oxidation state. However, this latter difference clearly reflects the in-solution behavior: only **1Pt–Glu** is sensitive^11^ to DMSO, a coordinating aprotic solvent, probably due to the aptitude of the equatorial neutral ligand ethene to act as a leaving group. The situation is reversed in the presence of water, a weaker ligand for platinum with respect to DMSO. In this condition, the Pt(IV)–carbene bond of **2Pt–Glu** is responsive (Scheme 2). It is plausible that the high formal charge present on the metal center favors the attack of OH^–^ ions that replace the hydrocarbyl ligand. This result is consistent with the poor selectivity toward cancer cells of **2Pt–Glu**, as the loss of the sugar fragment can have important effects on the internalization of the complex.

The stability of **1Pt–Glu** and **2Pt–Glu** toward hydrolysis is enhanced by ct-DNA and proteins: in the presence of these macromolecules, we had no evidence of structural variations of the complexes (Figures 10 and 11). Furthermore, both complexes retain their identity when interacting with DNA (Table 1), while cisplatin undergoes substitution of one chloride for other ligands. This finding confirms the higher general stability of coordinatively saturated species with respect to cisplatin. Although the two compounds show a diverse preference between the single and double strand of DNA, this difference does not offer the cue to clarify the clear difference in activity. A possible explanation can be found first by considering the different degree of internalization displayed by the complexes. The percentage of cellular uptake of **2Pt–Glu** is lower than that of either **1Pt–Glu** or cisplatin. This limitation is accompanied by the stability of the Pt(IV) complex in the presence of either glutathione or ascorbate, which are used on a regular basis to verify the tendency of platinum(IV) complexes to undergo reduction.^43^ These literature studies, carried out mainly on complexes containing Pt–Cl bonds, hypothesize that the efficient reduction occurs upon the formation of chloride bridges with the reductant, an event that neither **2Pt–Glu** nor its hydrolysis product **2Pt–OH** (Scheme 2) can give rise to. Therefore, on the basis of the established supposition that Pt(IV) agents need reduction to be effective, it can be assumed that another major reason for the poor activity of **2Pt–Glu** is the lack of reduction in the cytosolic environment; thus, no active species are able to initiate the cytotoxic process.

## Experimental Section

Reagents and solvents were purchased from Sigma-Aldrich and were used without further purification. NMR spectra were acquired on a 400 Bruker Avance Ultrashield 400 and on a 500 Varian Inova, located at the Dipartimento di Scienze Chimiche, Università di Napoli Federico II, Napoli (Italy). The solvents were CDCl_3_ (CHCl_3_, δ 7.26, and ^13^CDCl_3_, δ 77.0, as internal standards), (CD_3_)_2_SO ((CD_2_H)_2_SO), δ 2.49, as internal standard), D_2_O (HDO, δ 4.80 as internal standard), and CD_3_OD (CD_2_HOD, δ 3.30, ^13^CD^3^OD, δ 49.0, and ^195^PtCl_6_^2–^, δ 0, as internal standards). The following abbreviations were used for describing NMR multiplicities: s, singlet; d, doublet; dd, double doublet; triplet; app, apparent; m, multiplet; ABq, AB quartet; Me, methyl. Electrochemical measurements were recorded on a Reference 3000 Gamry instrument controlled by Framework software. Data analyses were performed with EChem Analyst electrochemical software. Precursors **2Pt–I**,^47^**Glu–Ag–Br**,^48,49^ and **Gal–Ag–Br**(11) were synthesized as described in the literature.

### Synthesis of **2Pt–Glu** and **2Pt–Gal**

The precursor **2Pt–I** (0.140 g, 0.256 mmol) was suspended into a solution of silver triflate (0.066 g, 0.256 mmol) in acetone (8 mL). After 10 min of stirring, AgI was filtered off on Celite. The filtrate was added to a solution of the appropriate **R–Ag–Br** (0.153 g, 0.256 mmol) in acetone (2 mL). The mixture was stirred and protected from light for 3 days at RT. Then, solid was filtered off, and the solvent was removed under vacuum, yielding a yellow oil. The product was obtained purified by SiO_2_ chromatography using 97:3 dichloromethane/methanol. **2Pt–Glu**: (yield 96%) ^1^H NMR, CDCl_3_, δ: 9.36 (m, 2H, H-2 phen and H-9 phen), 8.81 (d, 2H, H-4 and H-7 phen), 8.29(dd, 1H, H-3 or H-8 phen), 8.24 (dd, 1H, H-8 or H-3 phen), 8.22 (ABq, 2H, H-5 and H-6 phen), 7.00 (d, 1H, H-4 or H-5 imidazole), 6.82 (d, 1H, H-5 or H-4 imidazole), 5.58 (d, 1H, *J*_H1–H2_ = 8.6 Hz, H-1 glucose), 5.18 (m, 2H, H-2 and H-3 glucose), 5.06 (t, 1H, *J*_H4–H3_ = 9.8 Hz, H-4 glucose), 4.20 (m, 2H, H-6 and H-6′ glucose), 4.01 (m, 1H, H-5 glucose), 3.23 (s, 3H, Me imidazole), 2.11 (s, 3H, OAc), 2.10 (s, 3H, OAc), 1.98 (s, 3H, OAc), 1.20 (s, 3H, OAc), 1.41 (s, 3H, ^2^*J*_Pt–H_ = 70 Hz), 1.34 (s, 3H, ^2^*J*_Pt–H_ = 70 Hz), 0.08 (s, 3H, ^2^*J*_Pt–H_ = 55 Hz). ^13^C NMR, CDCl_3_, δ: 173.1 (*J*_Pt_ = 648 Hz), 170.5, 169.8, 169.5, 168.2, 148.0, 147.6, 146.0, 140.0, 139.4 (×2), 131.8 (×2), 128.9, 128.2, 126.7, 126.3, 125.3, 120.8 (q *J*_C–F_ = 322 Hz), 117.9, 83.7, 74.5, 72.4, 69.0, 68.2, 65.9, 61.6, 37.7, 20.7, 20.6, 20.4, 19.8, 5.3 (*J*_Pt_ = 506 Hz), −6.1 (*J*_Pt_ = 664 Hz), −6.8 (*J*_Pt_ = 664 Hz). ^195^Pt NMR, CD_3_OD, δ: −2777. Anal. Calcd (found): (C_34_H_41_F_3_N_4_O_12_PtS): C, 41.59 (41.81); H, 4.21 (4.26); N, 5.71 (5.67). **2Pt–Gal** (yield 98%) ^1^H NMR, CDCl_3_, δ: 9.34 (m, 2H, H-2 and H-9 phen), 8.84 (m, 1H, H-4 or H-7 phen), 8.80 (m, 1H, H-7 or H-4 phen), 8.28 (dd, 1H, H-3phen or H-8 phen), 8.24 (dd, 1H, H-8 or H-3 phen), 8.22 (s, 2H, H-5 and H-6 phen), 7.07 (d, 1H, H-4 or H-5 imidazole), 6.86 (d, 1H, H-5 or H-4 imidazole), 5.53 (d, 1H, *J*_H1–H2_ = 9.4 Hz, H-1 galactose), 5.49 (d, 1H, *J*_H4–H3_ = 3.2 Hz, H-4 galactose), 5.39 (t, 1H, *J*_H2–H3_ = 9.4 Hz H-2 galactose), 5.02 (dd, 1H, H-3 galactose), 4.13 (m, 2H, H-5 and H-6 galactose), 4.03 (m, 1H, H-6′ galactose), 3.20 (s, 3H, Me imidazole), 2.18 (s, 3H, OAc), 2.10 (s, 3H, OAc), 1.97 (s, 3H, OAc), 1.41 (s, 3H, ^2^*J*_Pt–H_ = 58 Hz), 1.36 (s, 3H, ^2^*J*_Pt–H_ = 88 Hz), 1.31 (s, 3H, OAc), 0.06 (s, 3H, ^2^*J*_Pt–H_ = 55 Hz). ^13^C NMR, CDCl_3_, δ: 172.9 (*J*_Pt_ = 640 Hz), 170.5, 169.9, 169.7, 168.4, 148.5, 147.8, 146.0, 139.7, 139.5, 131.8, 128.9 (×2), 128.3 (×2), 126.4, 126.2, 125.3, 124.0 (q *J*_C–F_ = 327 Hz), 118.7, 84.2, 73.5, 70.7, 67.3, 67.0, 61.3, 37.6, 20.8, 20.7, 20.4, 20.0, 5.4 (*J*_Pt_ = 508 Hz), −6.3 (*J*_Pt_ = 659 Hz), −6.6 (*J*_Pt_ = 658 Hz). ^195^Pt NMR, CD_3_OD, δ: −2789. Anal. Calcd (found): (C_34_H_42_F_3_N_4_O_12_PtS): C, 41.59 (41.75); H, 4.21 (4.10); N, 5.71 (5.83).

### Synthesis of **2Pt–Glu–dep**

The same procedure as above was adopted using methanol instead of acetone. The complex was purified by recrystallization from methanol/diethyl ether(yield 94%). ^1^H NMR, CD_3_OD, δ: 9.42 (m, 2H, H-2 phen and H-9 phen), 8.87 (m, 2H, H-4 and H-7 phen), 8.27 (s, 2H, H-5 and H-6 phen), 8.19 (dd, 1H, H-3 or H-8 phen), 8.14 (dd, 1H, H-8 and H-3 phen), 7.28 (d, 1H, H-4 or H-5 imidazole), 7.09 (d, 1H, H-5 or H-4 imidazole), 4.86 (d, 1H, *J*_H1–H2_ = 6.3 Hz, H-1 glucose), 3.63 (m, 1H, H-6 glucose), 3.49 (m, 1H, H-6′ glucose), 3.59 (s, 3H, Me imidazole), 3.25 (app t, 2H, H-2 and H-4 glucose), 2.71 (t, 1H, H-3 glucose), 2.66 (m, 1H, H-5 glucose), 1.46 (s, 3H, ^2^*J*_Pt–H_ = 69 Hz), 1.42 (s, 3H, ^2^*J*_Pt–H_ = 69 Hz), 0.03 (s, 3H, ^2^*J*_Pt–H_ = 53 Hz). ^13^C NMR, CD_3_OD, δ: 170.8 (*J*_Pt_ = 645 Hz), 148.9, 148.5, 146.1, 139.2, 139.1, 138.5, 131.9, 131.7, 128.0, 127.8, 126.2, 125.6, 124.4, 121.1 (q *J*_C–F_ = 322 Hz), 118.3, 85.8, 78.9, 76.5, 72.1, 68.9, 60.8, 36.8, 3.7 (*J*_Pt_ = 511 Hz), −7.1 (*J*_Pt_ = 663 Hz), −7.4 (*J*_Pt_ = 663 Hz). ^195^Pt NMR, CD_3_OD, δ: −2777. Anal. Calcd (found): (C_26_H_33_F_3_N_4_O_8_PtS): C, 38.38 (38.12); H, 4.09 (4.19); N, 6.89 (6.67).

### X-ray Crystallography

Single crystals of **2Pt–Gal** were obtained under slow diffusion of diethyl ether stratified on a dichloromethane solution of the complex at room temperature. Data were measured at room temperature using a Bruker-Nonius KappaCCD four-circle diffractometer (graphite monochromated Mo Kα radiation, λ = 0.71073 Å, CCD rotation images, thick slices, ϕ and ω scans to fill the asymmetric unit). It was not possible to collect data at low temperatures because crystals easily break under the cold N_2_ flux of the cryostream apparatus. The reduction of data and the semiempirical absorption correction were done using the SADABS program.^50^ The structure was solved by direct methods (SIR97 program^51^) and refined by the full-matrix least-squares method on *F*^2^ using the SHELXL-2018/3 program^52^ with the aid of the program WinGX.^53^ Anisotropic parameters were used for non-H atoms. All the H atoms were generated stereochemically and refined accordingly to the riding model with C–H distances in the range of 0.93–0.98 Å and *U*_iso_(H) equal to 1.2·*U*_eq_ of the carrier atom (1.5·*U*_eq_ for C_methyl_). Some acetate groups and triflate anions are affected by thermal disorder, which accounts for the rather high values of the displacement parameters. Some constraints were introduced in the last stage of the refinement to regularize the geometry and the displacement parameters using DFIX, SAME, SIMU, and DELU instructions of the SHELXL program. Disordered lattice solvent is present. It was not possible to model the disorder, and the PLATON SQUEEZE procedure was used to exclude the contribution of solvent to the structure. Details on crystal data and refinement parameters are reported in Table S1. The figures were generated using ORTEP-3^53^ and Mercury CSD 4.2^54^ programs.

### Spectrophotometric Measurements

UV–visible spectra of **2Pt–Glu** were collected on a Jasco V-650 UV–vis spectrophotometer at room temperature using 1 cm path length cuvettes. **2Pt–Glu** was first dissolved in pure DMSO and then diluted in the selected buffers at a final concentration of 50 μM. Spectra have been collected in 100% DMSO, in 10% DMSO/90% PBS (pH 7.4), and in 50% DMSO/50% PBS (pH 7.4) using the following setup: 240–450 nm wavelength range, 400 nm/min scanning speed, 2.0 nm bandwidth, and 1.0 nm data pitch. UV–vis measurements of **1Pt–Glu** and of **2Pt–Glu** with HEWL and HSA were performed by diluting the compounds’ stock solutions to 50 μM in 10% DMSO/90% PBS (pH 7.4). HEWL and HSA were added at about 17 μM to yield a final metal/protein ratio of 3:1. Spectra were recorded over 7 days at room temperature. The same procedure was used in order to collect UV–vis spectra of ct-DNA in the presence of the two platinum compounds. A solution of 10 μM ct-DNA was added to a 50 μM Pt solution containing 10% DMSO/90% PBS (pH 7.4). Spectra were collected over 7 days.

Fluorescence spectra were collected on a HORIBA Fluoromax-4 spectrofluorometer a 25 °C using a 1 cm path length cuvette. ct-DNA was incubated with EtBr in a 1:50 molar ratio for 30 min at room temperature. Then, the complex was diluted in 10 mM ammonium acetate buffer at pH 7.5 up to a ct-DNA final concentration of 200 μM. The ct-DNA–EtBr complex was then titrated with a **2Pt–Glu** solution at a concentration of 15 mM, and fluorescence emission spectra were recorded at an excitation at 545 nm. The spectra were registered after an equilibration time of 5 min following each addition. The CD spectra of ct-DNA were registered from 220 to 320 nm on a Jasco J-810 spectropolarimeter at 25 °C in the presence of different amounts of **2Pt–Glu**. Quartz cells with 0.1 cm path length were used. Each spectrum was obtained by averaging three scans and subtracting the contributions from the corresponding reference (10 mM ammonium acetate buffer at pH 7.5). Spectra were collected using samples obtained upon 24 h of incubation of ct-DNA with **2Pt–Glu** at 1:0.5, 1:1, and 1:2 molar ratios. ct-DNA concentration was 200 μM. Other experimental settings were as follows: 50 nm/min scan speed, 2.0 nm bandwidth, 0.2 nm resolution, 50 mdeg sensitivity, and 4 s response. The CD spectra of HSA and HEWL were registered upon a 24 h incubation in the presence of **1Pt–Glu** and **2Pt–Glu** at a 1:3 protein to metal molar ratio. Protein concentration was 0.10 mg/mL.

### Pt Compounds/DNA Interaction Studies by Electrospray Ionization Mass Spectrometry

A 20 mer double strand DNA (dsDNA) was obtained by an annealing procedure starting from two complementary single-stranded DNAs (ssDNAs) with nucleotide base sequences corresponding to 3′-CCA CCC GGA CCC CGT ACC TG-5′ for single strand 1 (ssDNA_1_) and to 3′-CAG GTA CGG GGT CCG GGT GG-5′ for single strand 2 (ssDNA_2_). The annealing reaction was carried out in water, mixing the single stranded oligonucleotides in an equal molar amount for 2 min at 95 °C and then cooling the mixture at room temperature for 45 min. **1Pt–Glu** and **2Pt–Glu** were dissolved in dimethyl sulfoxide (DMSO) (Bioshop, Burlington, ON, Canada) to a final concentration of 25 nmol/μL. Pt complexes were incubated with dsDNA in 10-fold molar excess at room temperature for 24 h. Sample mixtures were diluted 1:10 in 15 mM ammonium acetate buffer at pH 6.8; spectra were recorded in negative mode using a Q-Tof Premier (Waters, Milford, MA, USA) mass spectrometer. The acquisition was executed by direct injection at a 10 μL min^–1^ flow rate spanning the *m*/*z* range from 1000 to 3000. The capillary voltage was fixed to 2.7 kV, and source and desolvation gas temperatures were set to 70 °C. Raw data were processed by MassLynx 4.1 (Waters, Milford, MA, USA) software.

### Cytotoxicity and Uptake Experiments

Human A431 epidermoid carcinoma, murine BALB/c-3T3, and SVT2 fibroblasts were from ATCC. Human HaCaT keratinocyte cells were from Innoprot. Cells were cultured in Dulbecco’s modified Eagle’s medium (DMEM) (Sigma-Aldrich, St Louis, MO, USA) supplemented with 10% fetal bovine serum (HyClone), 2 mM l-glutamine, and antibiotics, all from Sigma-Aldrich, under a 5% CO_2_ humidified atmosphere at 37 °C. To test the cytotoxicity of **2Pt–Glu** and its derivatives, cells were seeded at a density of 2.5 × 10^3^ cells per well in 96-well plates. Twenty-four hours after seeding, increasing concentrations of compounds were added to the cells (0.1–125 μM). Cell viability was assessed by the MTT (3-(4,5-dimethylthiazol-2-yl)-2,5-diphenyltetrazolium bromide) assay after 48 h, as previously described.^11^ Cell survival was expressed as the percentage of viable cells in the presence of the Pt drug compared to the controls, represented by untreated cells and cells supplemented with identical volumes of DMSO (maximum 1% final volume). Each sample was tested in three independent analyses, each carried out in triplicate. To study the uptake of Pt drugs, A431 cells were incubated for 48 h in the presence of each drug, tested at the IC_50_ concentration. At the end of the incubation, Pt content was quantified by ICP-MS following a method previously reported.^55^ Briefly, Pt concentration was measured with three replicates using an Agilent 7700 ICP-MS instrument (Agilent Technologies) equipped with a frequency-matching radio frequency (RF) generator and third generation Octopole Reaction System (ORS3), operating with helium gas in ORF and the following parameters: RF power: 1550 W; plasma gas flow: 14 L min^–1^; carrier gas flow: 0.99 L min^–1^; He gas flow: 4.3 mL min^–1^. ^103^Rh was used as an internal standard (final concentration: 50 μg L^–1^). Standard solutions have been prepared in 5% nitric acid at four different concentrations (1, 10, 50, and 100 μg L^–1^).

### ^1^H NMR In-Solution Studies

**1Pt–Glu** and **2Pt–Glu** (10 mmol) were dissolved in DMSO-*d* (1 mL). The calculated volumes (60 μL) of the two solutions were diluted with the appropriate volume of 25 mM PBS buffer in D_2_O (pD 7.4) or DMSO-*d* to provide the final 1 mM concentration of the complex. Spectra were recorded at different times to evaluate the solution stability over 7 days.

Attempts of reduction were performed by adding ascorbic acid or glutathione to **2Pt–Glu** to afford solutions (600 μL of 10% DMSO-*d*/90% PBS (25 mM)) having 1 mM concentrations of the complex and 10–25 mM ascorbic acid or 2 mM glutathione. The solutions were incubated at 37 °C, and spectra were measured over time along 7 days.

The interaction with 2-deoxyguanosine monophosphate was studied by adding a dGMP solution in PBS (25 mM) to the appropriate volumes of **1Pt–Glu** and **2Pt–Glu** in DMSO-*d* to obtain final concentrations of 1 mM for the complexes and 4 mM for dGMP. Solutions were incubated at 37 °C, and spectra were recorded over 7 days.

### Electrochemical Studies

Electrochemical data were obtained by cyclic voltammetry (CV) and differential pulse voltammetry (DPV) under N_2_ at 20 °C using DMSO as solvent and [Et_3_MeN][BF_4_] (0.10 M) as supporting electrolyte. CV and DPV were performed in a three-electrode cell configuration consisting of a working glassy carbon (GC) electrode and two platinum wires as counter electrode and quasi-reference electrode. Prior to voltammetric experiments, the working electrode was polished with alumina, rinsed twice with water and acetone, and then dried. The analytes were introduced into the cell with a concentration of 1 mM. In CV, the scanning rate was 0.01 V s^–1^, and in DPV, the pulse size was 0.025 V. All potentials are referred to the ferrocene/ferrocenium(Fc/Fc^+^) couple.

## Conclusion

Here, we report the first direct comparison between the biological activities of Pt compounds with different oxidation states and almost indistinguishable structural features. Despite the evident similarity, the complexes have different properties, and the Pt(IV) species have been shown to be less cytotoxic than the corresponding Pt(II) compound. The reasons of the poor activity and selectivity displayed by the Pt(IV) complex have been investigated through several techniques by evaluating the structural and stability properties, cellular uptake, and the interaction with macromolecules. Similarities and differences between the two types of complexes have been disclosed and discussed in terms of their chemical properties.

Although the set of results were collected in vitro and therefore in conditions different from the complex living systems, they constitute pieces useful for reconstructing the colorful mosaic related to understanding the mechanism of action of platinum-based agents.

## Supporting Information Available

NMR spectra of the complexes, crystal data and additional molecular views, additional UV–vis and fluorescence spectra, ESI-MS data, CV and DPV voltammograms, and reduction peaks (PDF)

## Accession Codes

CCDC 1972544 contains the supplementary crystallographic data for this paper. These data can be obtained free of charge via www.ccdc.cam.ac.uk/data_request/cif, or by emailing data_request@ccdc.cam.ac.uk, or by contacting The Cambridge Crystallographic Data Centre, 12 Union Road, Cambridge CB2 1EZ, UK; fax: +44 1223 336033.
