# Supplementary material for: Pt(II) versus Pt(IV) in Carbene Glycoconjugate Antitumor Agents: Minimal Structural Variations and Great Performance Changes
Source: Inorg Chem. 2020 Mar 4;59(6):4002–14. doi: 10.1021/acs.inorgchem.9b03683 (PMC7997382; doi:10.1021/acs.inorgchem.9b03683)
Supplement: Supplementary file 1 — ic9b03683_si_001.pdf [file ic9b03683_si_001.pdf]

Supporting Information

Pt(II) versus Pt(IV) in Carbene Glycoconjugate Antitumor Agents: Minimal Structural Variations and Great Performance Changes

Alfonso Annunziata,[a] Angela Amoresano,[a] Maria Elena Cucciolito,[a,b] Roberto Esposito,[a,b] Giarita Ferraro,[c] Ilaria Iacobucci,[a] Paola Imbimbo,[a] Rosanna Lucignano,[a] Massimo Melchiorre,[d] Maria Monti,[a] Chiara Scognamiglio,[a] Angela Tuzi,[a] Daria Maria Monti,[a,] Antonello Merlino,[a,]* Francesco Ruffo[a,b,]**

[a]Dipartimento di Scienze Chimiche, Università di Napoli Federico II, Complesso Universitario di Monte S. Angelo, via Cintia 21, 80126 Napoli (Italy). [b]CIRCC, via Celso Ulpiani 27, 70126 Bari (Italy). [c]Dipartimento di Chimica Ugo Schiff, Università di Firenze, Sesto Fiorentino, FI 50019 (Italy). [d]ISUSCHEM, piazza Carità 32, 60134 Napoli (Italy)

Figure S1. ^{1}H and ^{13}C NMR spectra of **2Pt-Glu** (in $CDCl_3$, 400 MHz, 298 K).

Figure S2. ^{1}H and ^{13}C NMR spectra of **2Pt-Gal** (in $CDCl_3$, 400 MHz, 298 K).

Figure S3. ^{1}H and ^{13}C NMR spectra of **2Pt-Glu-dep** (in CD_3OD, 400 MHz, 298 K).

Figure S4. ^{195}Pt NMR spectra of **2Pt-Glu**, **2Pt-Glu-dep**, **2Pt-Gal**, **2Pt-OH** (in CD_3OD, 298 K).

Table S1. Crystal data and structure refinement details for **2Pt-Gal**.

Figure S5. Ortep view of **2Pt-Gal** with thermal ellipsoid drawn at 30% probability level.

Figure S6. Superimposition of **2Pt-Gal** and **1Pt-Glu**.

Figure S7. Small bowlike distorsion of phenantroline plane.

Figure S8. Projection of **2Pt-Gal** in the edge of the coordination plane showing the not flat shape of galactosyl group.

Figure S9. Crystal packing of **2Pt-Gal** viewed along **a** axis.

Figure S10. Time course UV-vis spectra of 50 μM **2Pt-Glu** in 50% DMSO – 50% PBS pH 7.4 (B).

Figure S11. ^{1}H spectrum of **2Pt-Glu** in 10% DMSO-d – 90% PBS-d pH 7.4 after 24 h.

Figure S12. Fluorescence emission spectra of DNA-EtBr complex upon titration with a solution of 15 mM of **2Pt-Glu**.

Figure S13. ESI-MS spectrum of dsDNA incubated with cisplatin (cisPt).

Table S2. Results of ESI-MS analysis of species formed upon reaction of DNA with CisPt.

Figure S14. Time course UV-vis spectra of 50 μM **1Pt-Glu** (A-B) and 50 μM **2Pt-Glu** (C-D) in 10% DMSO – 10 mM sodium acetate buffer pH 4.0 in the presence of HEWL in a 1:3 protein to metal molar ratio.

Figure S15. CV voltammograms of **2Pt-Glu** and **2Pt-OH** (1mM in DMSO-0.10 M $[Et_3MeN][BF_4]$).

Figure S16. DPV voltammograms of **2Pt-Glu** and **2Pt-OH** (1mM in DMSO-0.10 M $[Et_3MeN][BF_4]$).

Table S3. Reduction peaks of **2Pt-Glu** and **2Pt-OH** (1mM in DMSO-0.10 M $[Et_3MeN][BF_4]$).

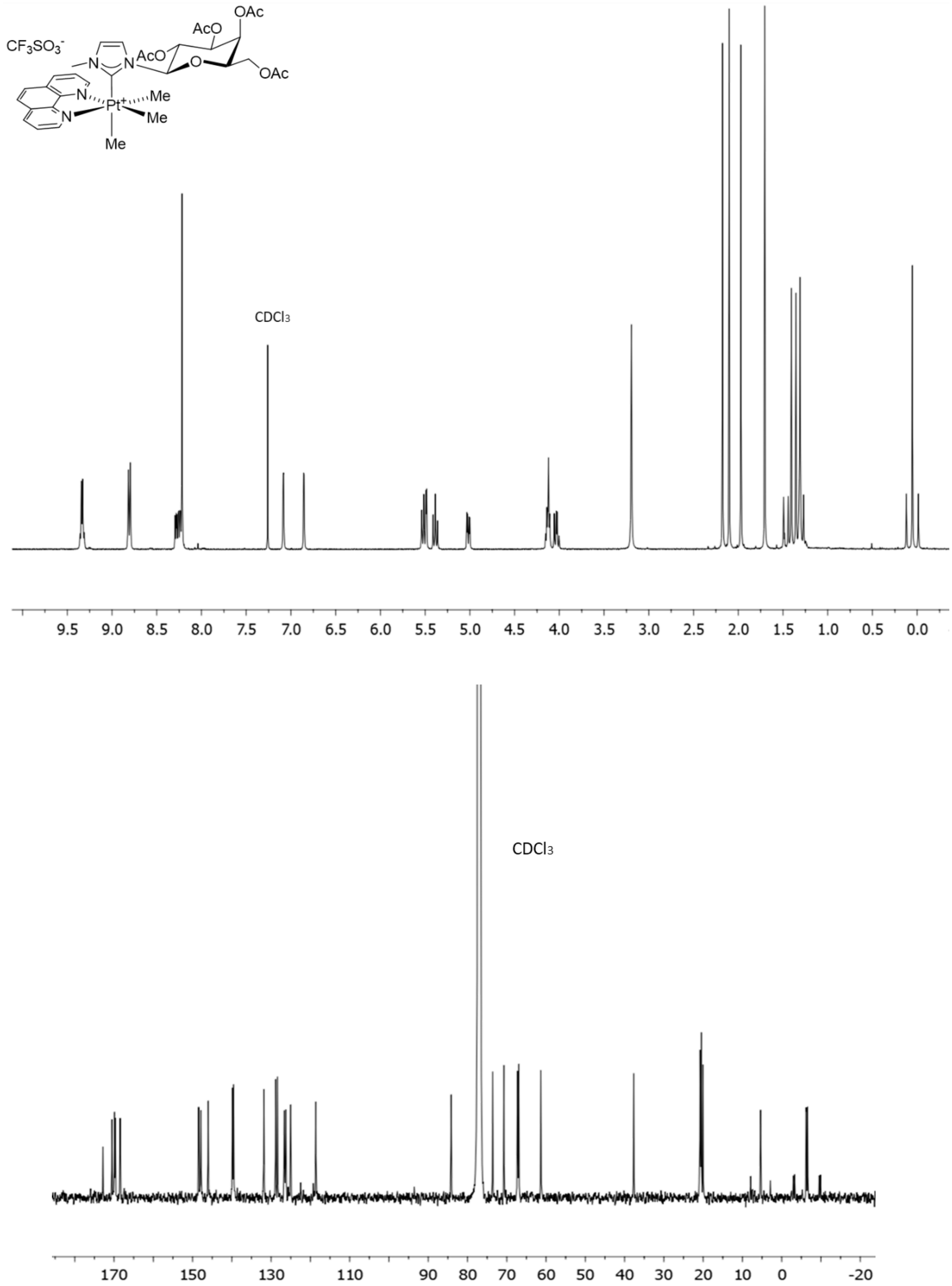

Figure S1. ^{1}H and ^{13}C NMR spectra of **2Pt-Glu** (in $CDCl_3$, 400 MHz, 298 K).

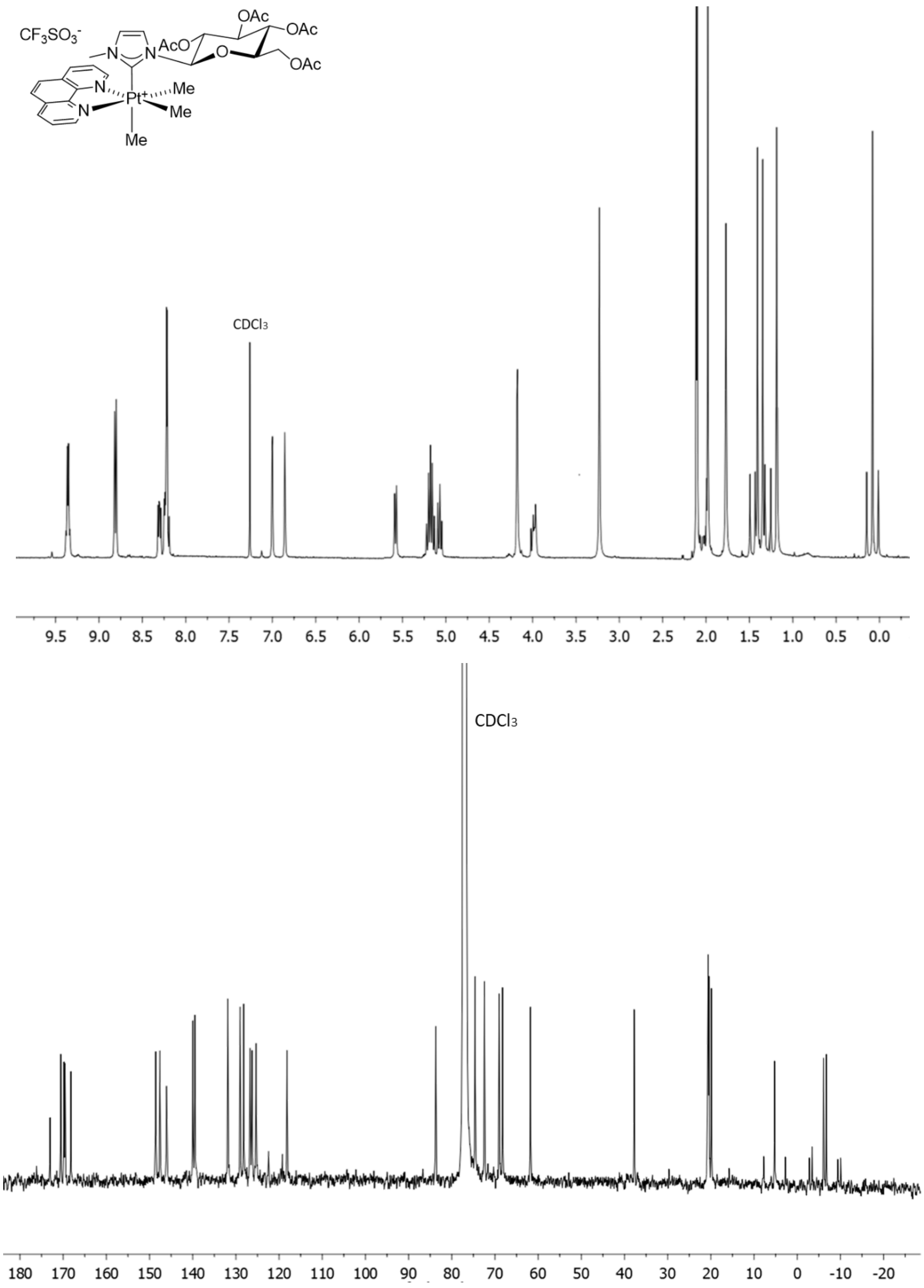

Figure S2. ^{1}H and ^{13}C NMR spectra of **2Pt-Gal** (in $CDCl_3$, 400 MHz, 298 K

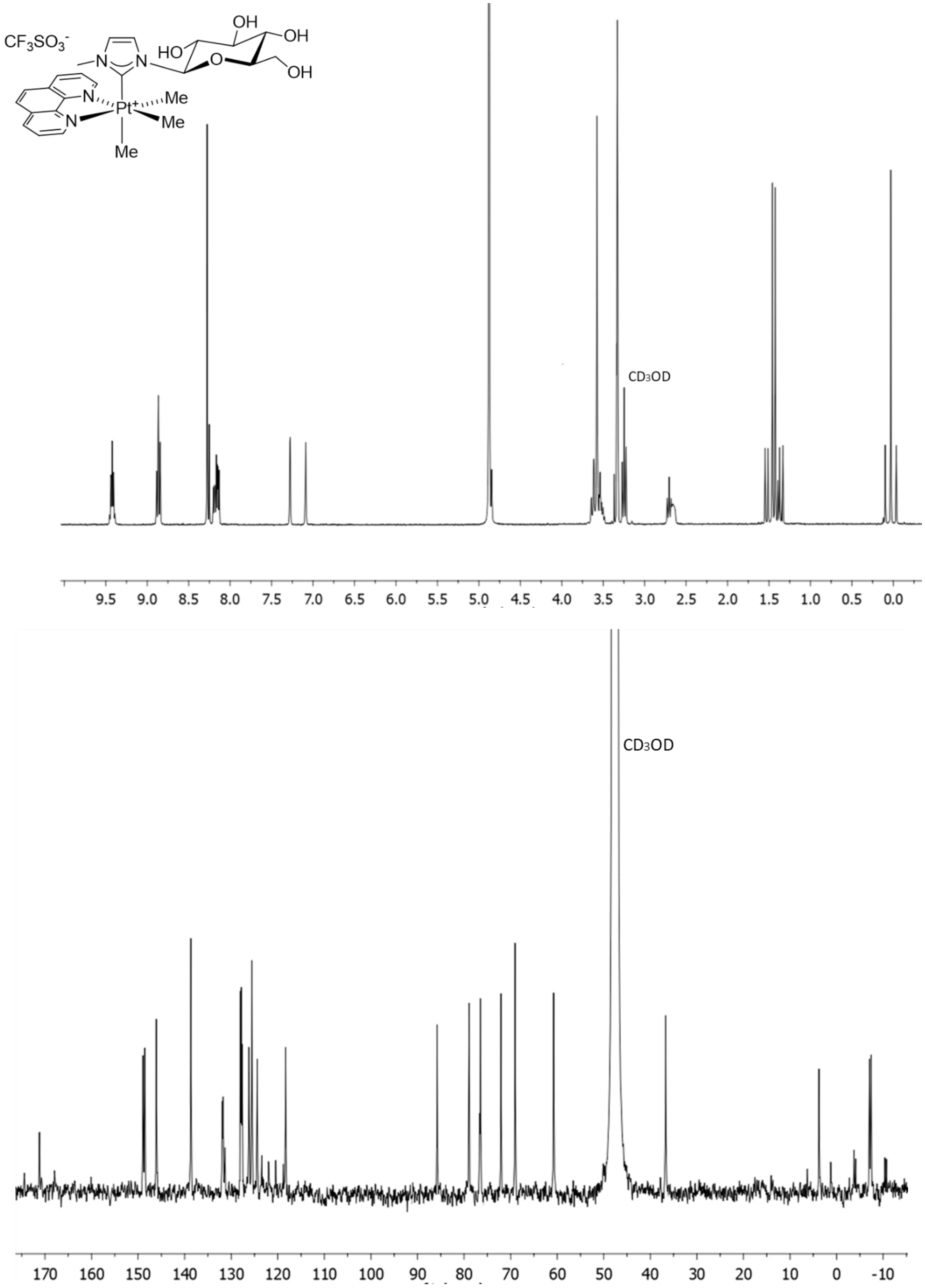

Figure S3. ^{1}H and ^{13}C NMR spectra of **2Pt-Glu-dep** (in CD_3OD, 400 MHz, 298 K).

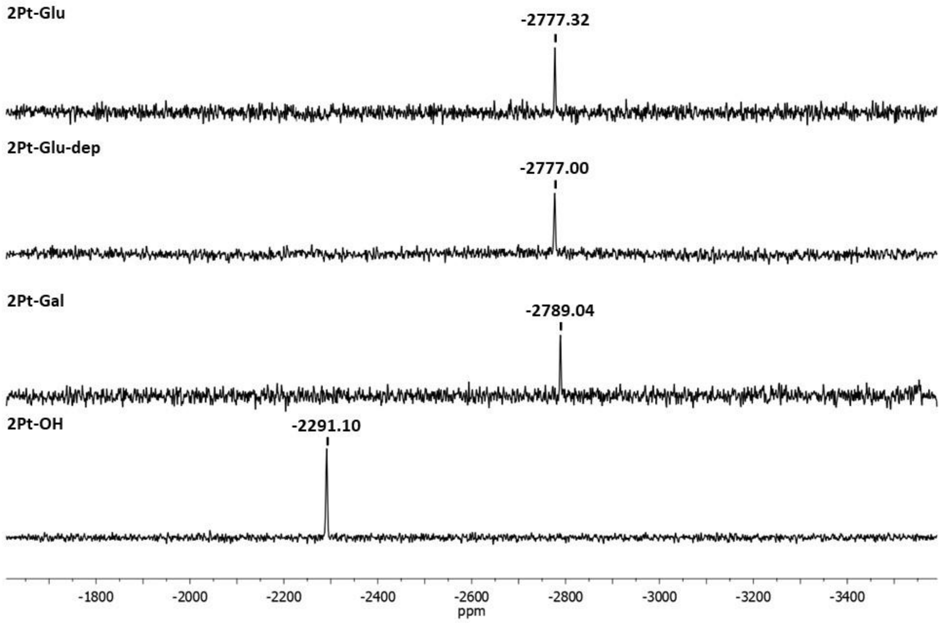

Figure S4. ^{195}Pt NMR spectra of **2Pt-Glu**, **2Pt-Glu-dep**, **2Pt-Gal**, **2Pt-OH** (in CD_3OD, 298 K).

Table S1 Crystal data and structure refinement details for **2Pt-Gal**

Empirical formula	C33 H41 N4 O9 Pt · C F3 O3 S
Formula weight	981.86
Temperature	298(2)K
Wavelength	0.71073 Å
Crystal system, space group	Triclinic, P 1
Unit cell dimensions	a = 13.8920(14)Å, α = 74.640(11)°
	b = 18.426(2)Å, β = 73.436(8)°
	c = 19.187(2)Å, γ = 68.649(9)°
Volume	4312.5(9)Å3
Z, Calculated density	4, 1.512 Mg/m3
Absorption coefficient	3.374 mm-1
F(000)	1960
Crystal size	0.40 x 0.30 x 0.15 mm
Theta range for data collection	2.150 to 27.500 °
Reflections collected / unique	47334 / 27957 [R(int) = 0.0418]
Data / restraints / parameters	27957 / 217 / 2012
Goodness-of-fit on F2	1.072
Final R indices [I>2sigma(I)]	R1 = 0.0533, wR2 = 0.1316
R indices (all data)	R1 = 0.0876, wR2 = 0.1555
Absolute structural parameter	0.096(11)
Largest diff. peak and hole	1.262 and -1.307e·Å-3

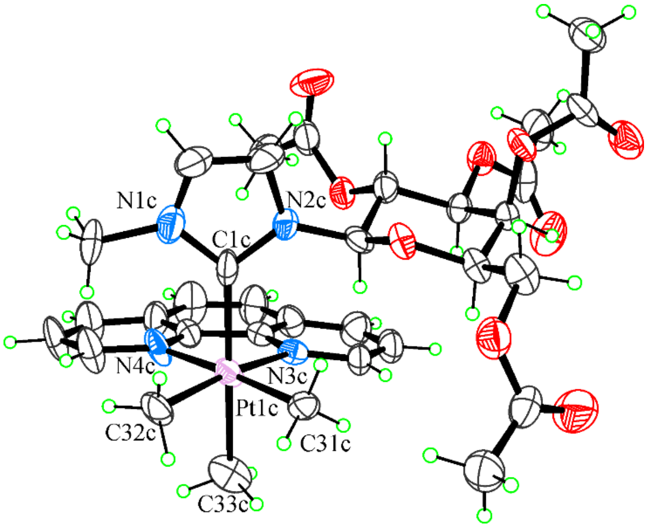

Figure S5. Ortep view of **2Pt-Gal** with thermal ellipsoid drawn at 30% probability level. Selected bond lengths and angles: Pt1c-C1c = 2.13(2), Pt1c-N3c = 2.175(14), Pt1c-N4c = 2.195(17) , Pt1c-C31c = 2.021(17), Pt1c-C32c = 2.04(2), Pt1c-C33c = 2.10(2). Å; N3c-Pt1c-N4c = 77.7(7), C31c-Pt1c-C32c = 83.0(10), C32c-Pt1c-N4c = 98.9(9), C31c-Pt1c-N3c = 100.3(8), C33c-Pt1c-C1c = 176.1(9) °.

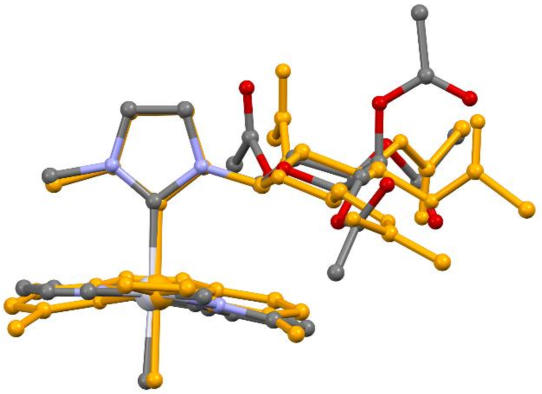

Figure S6. Superimposition of **2Pt-Gal** (element color, A-label molecule) and **1Pt-Glu** (orange, ref. 11 of manuscript). Ball-and-stick model, H atoms not drawn for clarity.

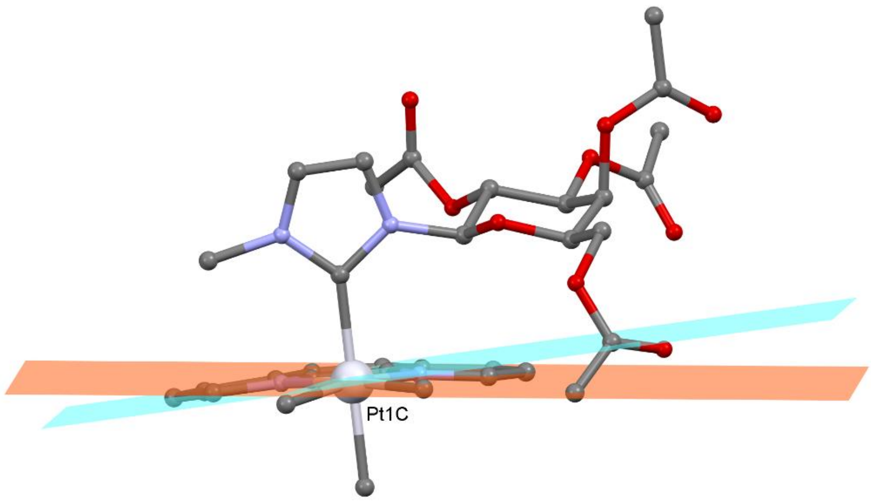

Figure S7. Small bowlike distorsion of phenantroline plane (C-label molecule). The angle between the mean planes of outer rings is 8(2)°.

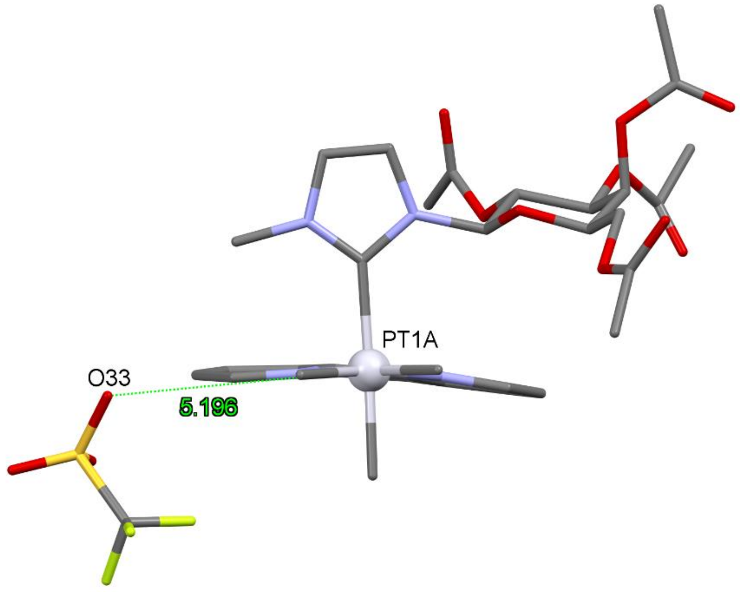

Figure S8. Projection of **2Pt-Gal** (A-label molecule) in the edge of the coordination plane showing the not flat shape of galactosyl group. The shortest distance Pt···O(triflate) is reported as a green dashed line. Hydrogen atoms are not shown for clarity.

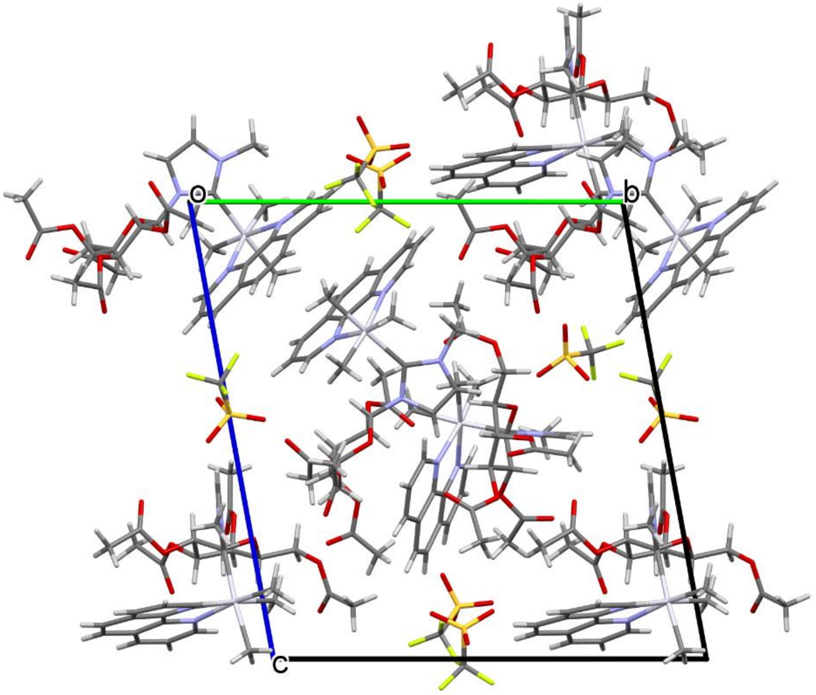

Figure S9. Crystal packing of **2Pt-Gal** viewed along **a** axis.

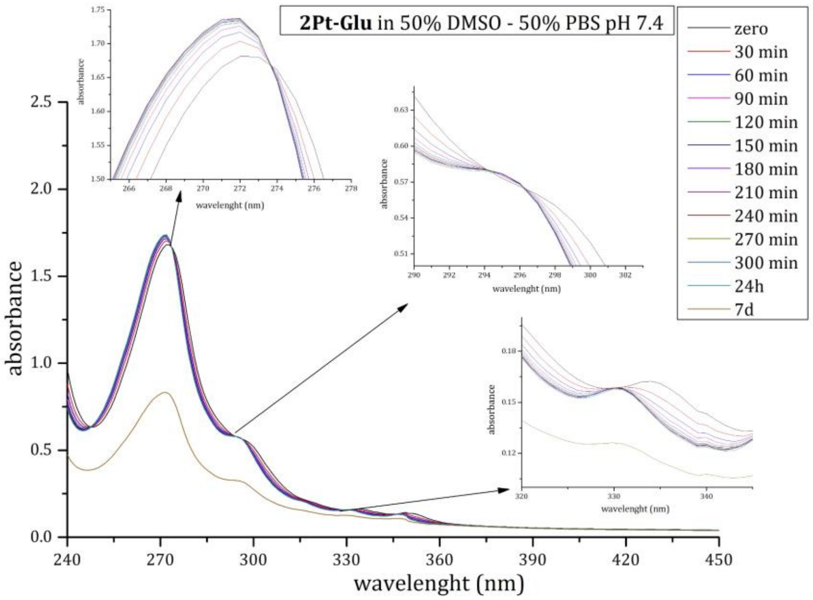

Figure S10. Time course UV-vis spectra of 50 μM **2Pt-Glu** in 50% DMSO – 50% PBS pH 7.4 (B). Details of the isosbestic points are also shown.

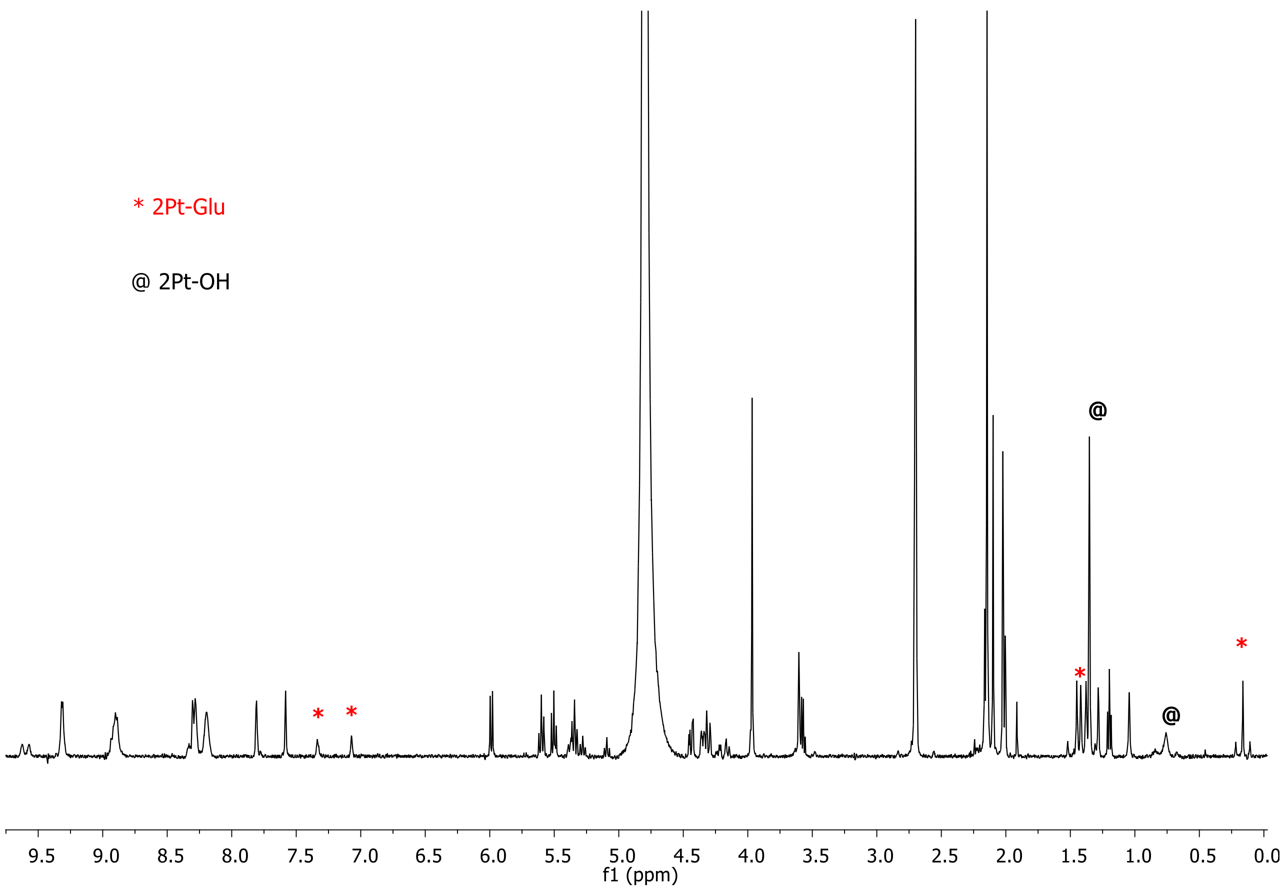

Figure S11. ^{1}H spectrum of **2Pt-Glu** in 10% DMSO-d – 90% PBS-d pH 7.4 after 24 h.

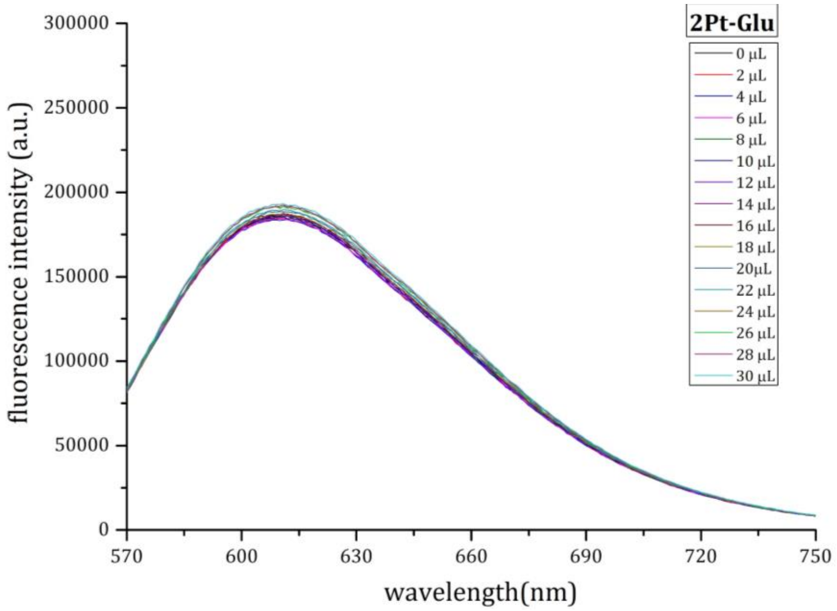

Figure S12. Fluorescence emission spectra of DNA-EtBr complex upon titration with a solution of 15 mM of **2Pt-Glu**.

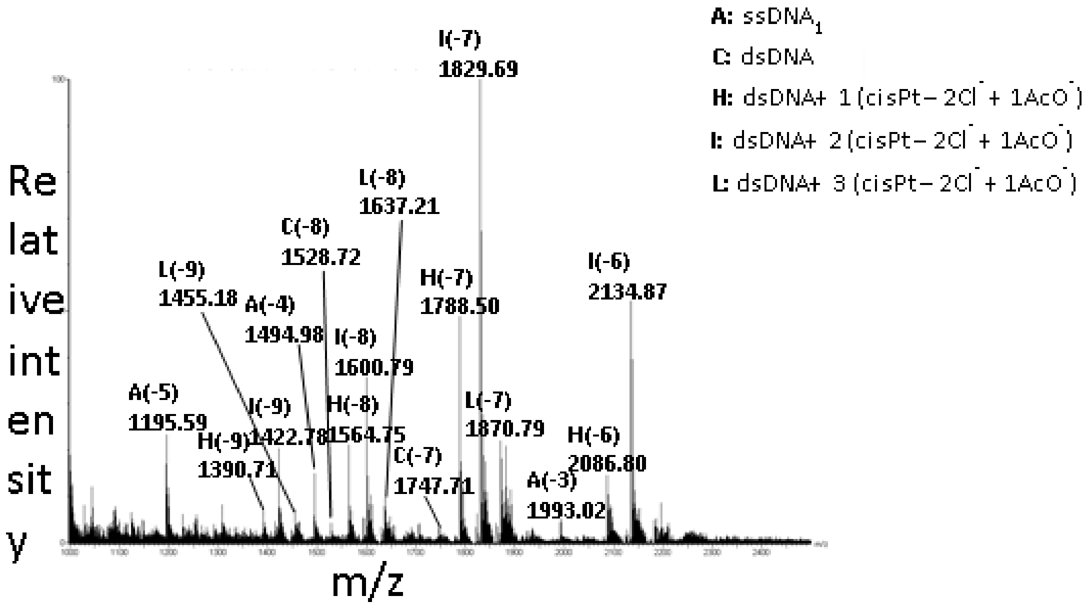

Figure S13. ESI-MS spectrum of dsDNA incubated with cisplatin (cisPt).

Metal complex	Signal (m/z)	Signal charge	Exp MW (Da)	Theoretical MW (Da)	Species
Cis-Pt	1195.59<br>1494.98<br>1993.02	A (-5)<br>A (-4)<br>A (-3)	5983.32±0.46	5983.9	$ssDNA_1$
	1528.72<br>1747.71<br>2038.64	C (-8)<br>C (-7)<br>C (-6)	12237.82±0.06	12239	dsDNA
	1390.71<br>1564.75<br>1788.5<br>2086.8	H (-9)<br>H (-8)<br>H (-7)<br>H (-6)	12526.23±0.52	12527.18	dsDNA + 1(cisPt - 2Cl^- +1AcO^-)
	1422.78<br>1600.79<br>1829.69<br>2134.87	I (-9)<br>I (-8)<br>I (-7)<br>I (-6)	12814.67±0.45	12815.36	dsDNA + 2(cisPt - 2Cl^- +1AcO^-)
	1455.18<br>1637.21<br>1870.79	L (-9)<br>L (-8)<br>L (-7)	13104.69±1.47	13103.54	dsDNA + 3(cisPt - 2Cl^- + 1 AcO^-)

Table S2. Results of ESI-MS analysis of species formed upon reaction of DNA with CisPt. The m/z values detected in MS spectra and their relative charges, as well as experimental (Exp) and theoretical (Theor) monoisotopic mass values and the corresponding ion species are reported. dsDNA = double stranded DNA; ssDNA = single stranded DNA; cisPt= cisplatin; AcO^- = acetate ion; Cl^- = chloride ion.

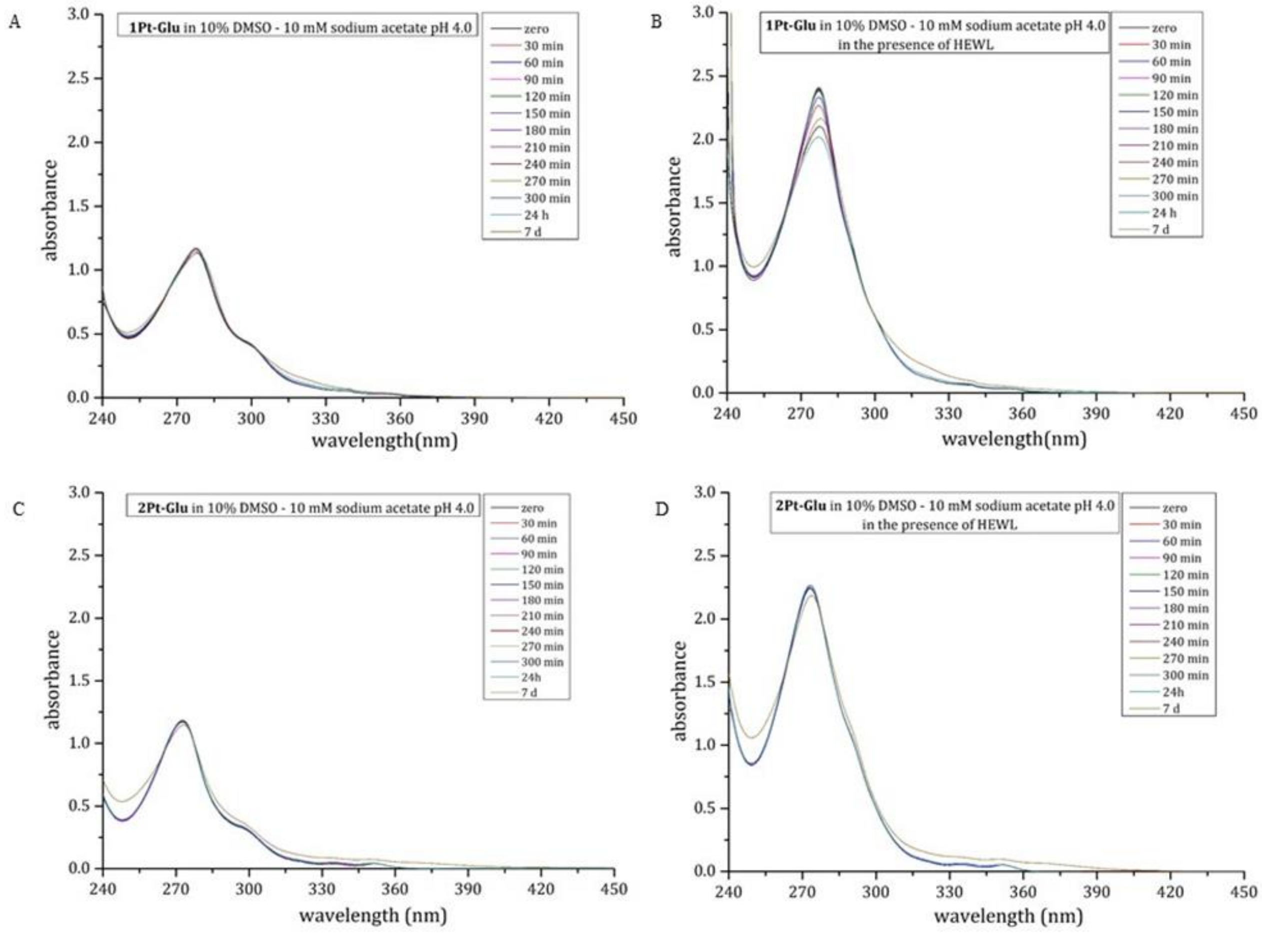

Figure S14. Time course UV-vis spectra of 50 μM **1Pt-Glu** (A-B) and 50 μM **2Pt-Glu** (C-D) in 10% DMSO – 10 mM sodium acetate buffer pH 4.0 in the presence of HEWL in a 1:3 protein to metal molar ratio.

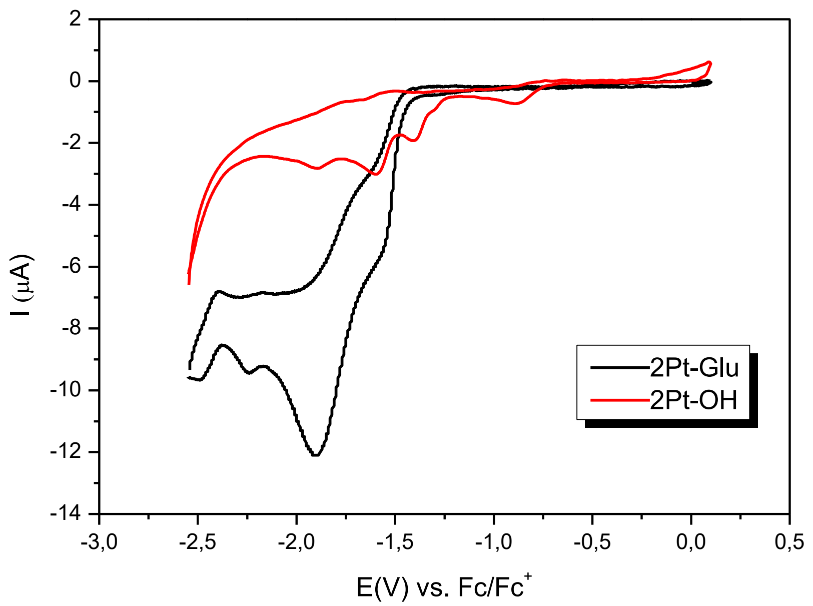

Figure S15. CV voltammograms of **2Pt-Glu** and **2Pt-OH** (1mM in DMSO-0.10 M $[Et_3MeN][BF_4]$).

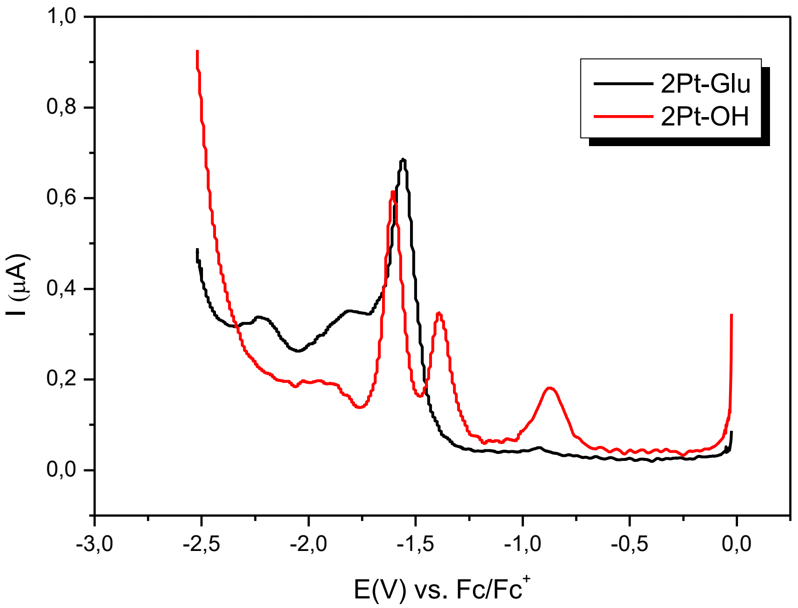

Figure S16. DPV voltammograms of **2Pt-Glu** and **2Pt-OH** (1mM in DMSO-0.10 M $[Et_3MeN][BF_4]$).

Table S3. Reduction peaks of **2Pt-Glu** and **2Pt-OH** (1mM in DMSO-0.10 M $[Et_3MeN][BF_4]$).

	E_{red1}	E_{red2}	E_{red3}	E_{red4}
**2Pt-Glu**	-1.54	-1.89	/	/
**2Pt-OH**	-0.89[b]	-1.40	-1.59	-1.89

[a]1-methyl-3-(2,3,4,6-tetra-*O*-acetyl-β-D-glucopyranosyl)imidazolylidene bromide and 1,10-phenanthroline are not electroactive above -2 V. [b]Attributed to the reduction of O-H.
